# The Sommersdorf mummies—An interdisciplinary investigation on human remains from a 17th-19th century aristocratic crypt in southern Germany

**DOI:** 10.1371/journal.pone.0183588

**Published:** 2017-08-31

**Authors:** Amelie Alterauge, Manuel Kellinghaus, Christian Jackowski, Natallia Shved, Frank Rühli, Frank Maixner, Albert Zink, Wilfried Rosendahl, Sandra Lösch

**Affiliations:** 1 Department of Physical Anthropology, Institute of Forensic Medicine, University of Bern, Bern, Switzerland; 2 Institute of Pre- and Protohistory and Near Eastern Archaeology, University of Heidelberg, Heidelberg, Germany; 3 Institute of Forensic Medicine, University of Bern, Bern, Switzerland; 4 Institute of Evolutionary Medicine, University of Zurich, Zurich, Switzerland; 5 Institute for Mummy Studies, Eurac Research, Bolzano, Italy; 6 German Mummy Project, Reiss-Engelhorn-Museen, Mannheim, Germany; Seoul National University College of Medicine, REPUBLIC OF KOREA

## Abstract

Sommersdorf Castle (Bavaria, Germany) is a medieval castle complex which has been inhabited by the aristocratic family *von Crailsheim*. The deceased were entombed in a crypt located in the parapets underneath the castle’s church, resulting in mummification of the bodies. Based on the family chronicle and oral history, identities have been ascribed to the mummies. The aim of the study is therefore to test the accuracy of the historical records in comparison to archaeological, anthropological and genetic data. Today, the crypt houses eleven wooden coffins from the 17^th^ to 19^th^ century AD. In ten of these, mummified and scattered human remains were found. Archive records were studied in order to identify names, ancestry, titles, occupation, date of birth and death, and place of interment of the individuals. The coffins were visually inspected and dated by typo-chronology, and the mummified and scattered skeletal remains were subjected to a physical anthropological examination. In total, the crypt contains the remains of a minimum number of nine individuals, among them three adult males, five adult females and one infant. A detailed scientific examination, including prior conservation, ancient DNA analyses, and computed tomography (CT), was performed on five mummies. By means of the CT data age at death, sex, body height, pathologies, and anatomical variants were investigated. CT analysis further showed that the bodies were naturally mummified. Mitochondrial DNA analyses revealed that the tested individuals are not maternally related. In addition, health, living conditions and circumstances of death of the entombed individuals could be highlighted. Being confronted with the strengths, weaknesses and limitations of each methodological approach, probable identification was achieved in two cases.

## Introduction

During the early modern period, Europe’s social, economic and religious elite intended to reflect and legitimise their status in life also during funeral. This attitude had been intensified by the Reformation leading to a stronger emphasis on the individual and thereby also on the individual death. Elaborate funeral rites with services, sermons and music were conducted to accompany the dead to his final resting place [1]. Special burial places inside or underneath churches that were linked to dynastic families were reserved for stone sarcophagi and metal coffins [2]. But crypt burial was not only the privilege of the sovereigns but also of the high and middle class, such as the clergy, the nobility, and the bourgeoisie. Due to favourable environmental conditions, the inventory of such crypts can be preserved, including the coffins, the clothes, and the deceased. The identity of the entombed individuals is usually known through inscriptions on the coffin itself or through historical records providing information on the life history of the deceased. Therefore, crypt burials offer the unique opportunity for multidisciplinary research to study the physical remains of the elites and their perceptions of death and burial.

Interdisciplinary investigations of post-medieval church crypts have recently been undertaken by different specialists in order to synoptically compare the archaeological, historical, anthropological and ancient DNA records [3–10]. However, the available biographical data are not always sufficient to unambiguously correlate historical records with the preserved human remains, especially when looters have disturbed the integrity of the coffins.

Sommersdorf Castle is located south of the city of Ansbach in Bavaria (Germany) and was erected in the late 14^th^ century AD. It is a moated castle complex with defensive walls, a keep and a former drawbridge. Outside the castle itself are several subsidiary buildings, including the protestant castle church of St. Stephen and Sebastian [11]. Since 1550, the castle has been inhabited by members of the aristocratic family *von Crailsheim* who were advisers and executive officers to the margraves of Brandenburg-Ansbach [12]. Even today, members of the Crailsheim family are still living in the castle.

**Fig 1:**
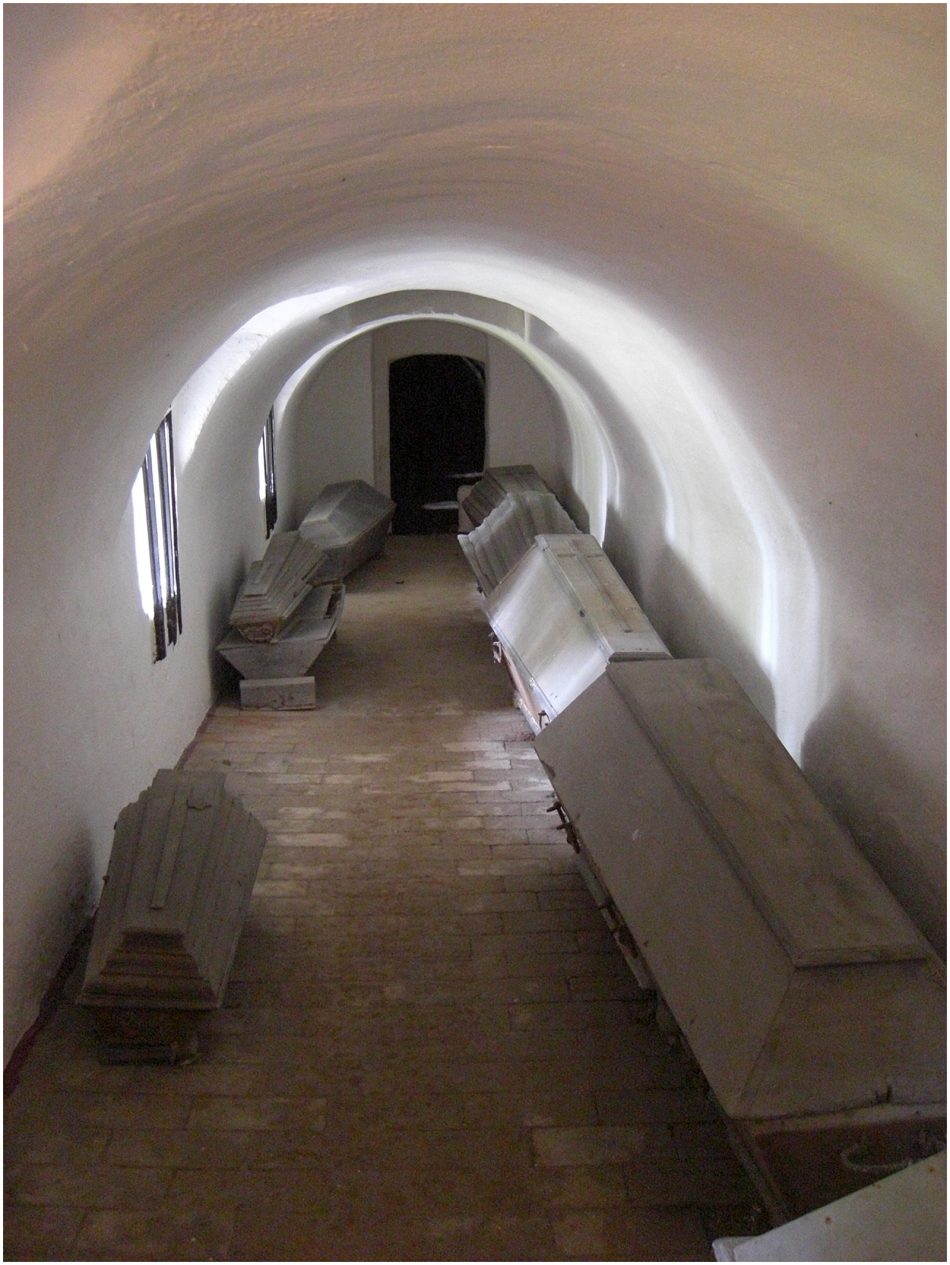
Crypt of Sommersdorf castle in May 2014, view from the eastern entrance to the west. The following coffins are visible on the figure: coffins no. 1–5 (right), no. 10, 7, 8, 9, 11 (left). For former position of the coffins see Fig 2. Reprinted from [59] under a CC BY license, with permission from the Society for Historical Archaeology, original copyright A. Alterauge, 2014.

**Fig 2:**
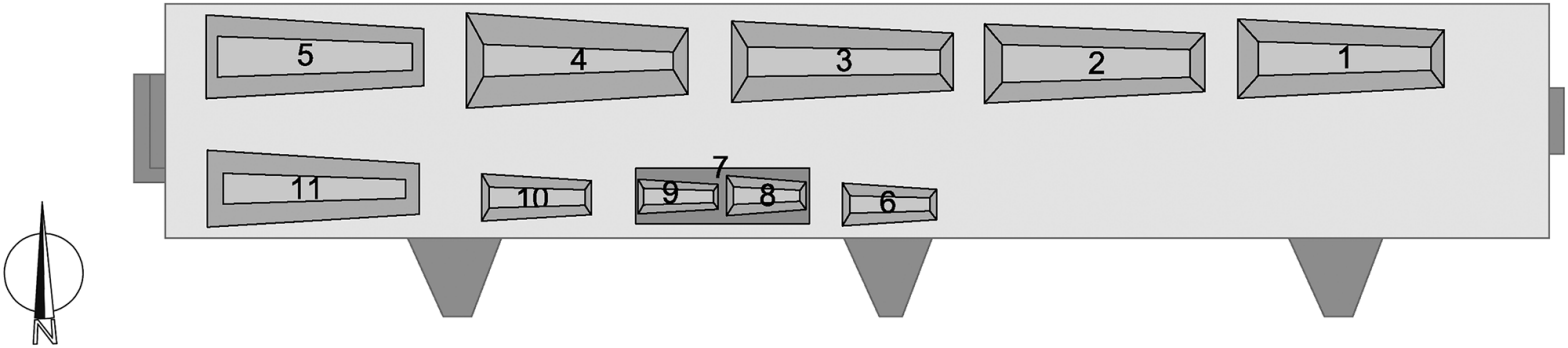
Position and numbering of the coffins in the crypt in September 2013 (plan: A. Alterauge).

Parts of the parapets underneath the church were re-used as a family crypt from the 17^th^ to the 19^th^ century AD (Figs 1 and 2). Due to previous lootings, the exact number of inhumations in the crypt is unknown.

As the bodies are mummified, the mummies from Sommersdorf have turned into objects of public interest in the past, and “stories” about their identity have arisen. However, these identifications were mainly based on the family chronicle [13] and oral history and were later uncritically adopted [14, 15].

The present study was initiated in the framework of the German Mummy Project (Reiss-Engelhorn-Museen, Mannheim, Germany) prior to an international exhibition project [16, 17]. It involved conservation, computed tomography, ancient DNA analyses, archaeological and anthropological examination and historical research on the individuals. First investigations were already carried out on a sub-sample of the Sommersdorf mummies and have focused on specific variants and pathologies [15, 18].

The purpose of the study is to test the accuracy of primary and secondary historical sources in comparison to archaeological, anthropological and genetic data. Thereby, the ascribed identifications are questioned.

## Material

Today, the crypt contains eleven coffins (Table 1). Six are adult coffins (no. 1–5, 11), four are children coffins (no. 6, 8–10), and one is only a coffin lid (no. 7) (Fig 2). All the adult coffins contain mummified remains whereas only one child mummy is preserved (no. 9). Coffin no. 7 contains the partially mummified remains of an adult individual. Coffin no. 8 is empty while scattered skeletal elements are found within the coffins no. 5, 6 and 10.

**Table 1:** Content of the coffins and performed analyses. The individuals C and G were sampled twice (*) for mtDNA analysis.

Coffin no.	Coffin length (cm)	Ind.	Content	Special features	Restoration	CT	mtDNA analysis (sample)
**1**	188	A	mummy	hair, stockings, gloves	yes	yes	yes (cervical vertebra)
**2**	201	B	mummy	boots, gloves	yes	yes	no
**3**	202	C	mummy		yes	yes	yes (rib & diaphragm)*
**4**	202	D	mummy	hair, stockings	yes	yes	yes (bone/tooth)
**5**	198	E	mummy (+ supernumerary scattered skeletal elements)		no	no	yes (bone)
**6**	86		scattered skeletal elements		no	no	no
**7**	158	F	partial mummy		no	no	no
**8**	72	-					
**9**	73	G	mummy		yes	yes	yes (rib)*
**10**	100		scattered skeletal elements		no	no	no
**11**	193	H	mummy	stockings, gloves	no	no	no

no. = number; ind. = individual; CT = computed tomography; mtDNA = mitochondrial DNA

In total, eight mummies could be investigated. The mummies are in a good condition even though clothes are mostly missing except for gloves, stockings and boots.

Restoration, computed tomography (CT) and ancient DNA (aDNA) analysis were performed on those mummies selected for the exhibition and additionally on those with specific research questions (e.g., cause of death).

The available historical records comprise the death register of Sommersdorf parish (after 1678) as well as several archival documents as primary sources [19] (S1 File). While primary historical sources are contemporary records, secondary sources build upon the primary ones and set the obtained information in a new context.

**Fig 3:**
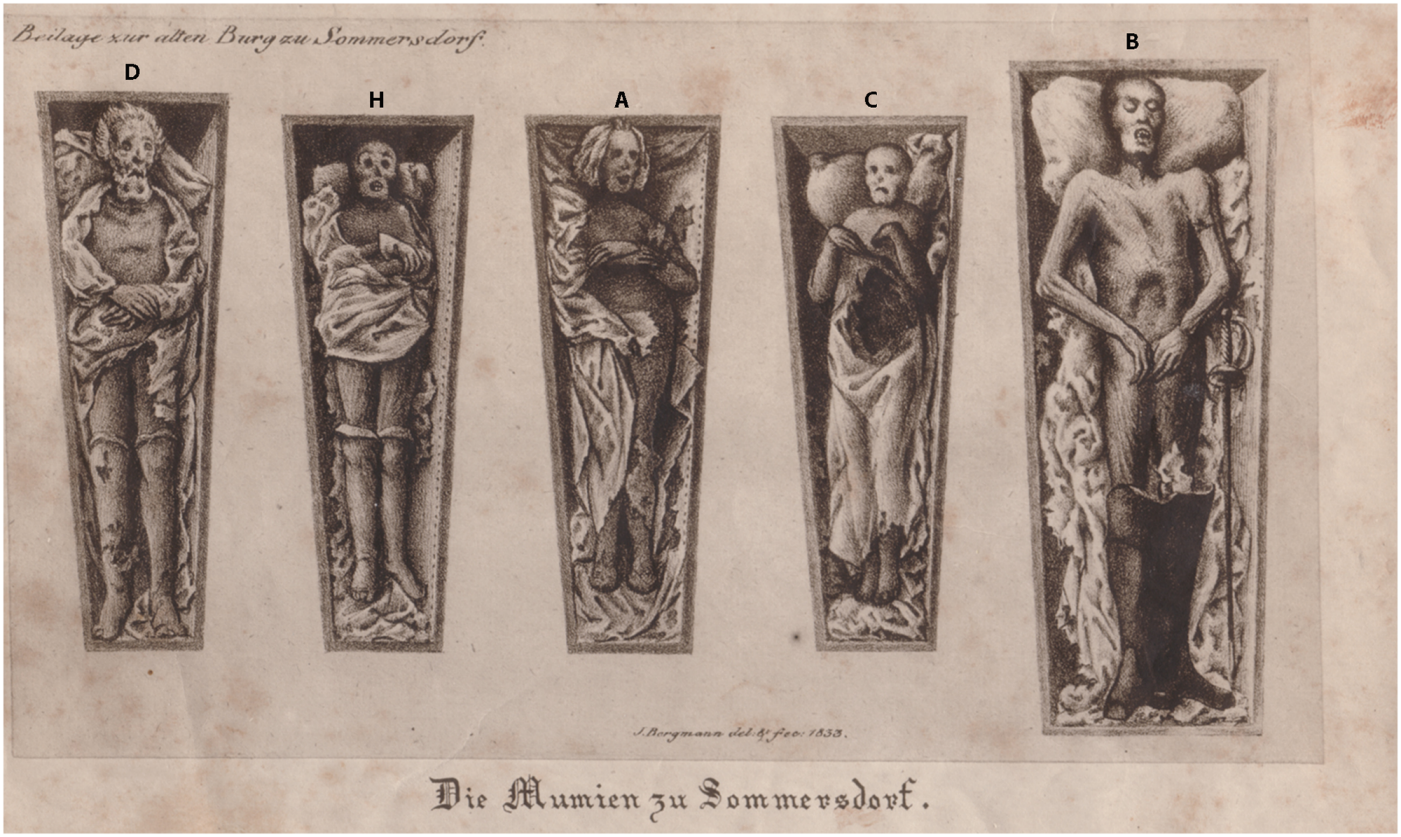
Reproduction of a 1833 lithograph by Josef Bergmann, an artist from Nuremberg, showing five mummies from the crypt of Sommersdorf castle. The labelling corresponds to the mummies’ alphabetical identifiers in this study. Adapted and reprinted from [59] under a CC BY license, with permission from the Society for Historical Archaeology, original copyright M. Freiherr von Crailsheim, 2007.

As a secondary source, the family chronicle summarizes the history of the barons *von Crailsheim* and lists the lineage and place of burial of each family member [13]. Those data are complemented by genealogical handbooks [20, 21]. A lithograph from Josef Bergmann (1795–1842) portrays the preservation of five mummies in 1833 (Fig 3) [22].

## Methods

As the crypt is located on private property, access was allowed and ensured by the current inhabitant of the castle who is a descendant of the Crailsheim family branch. All necessary permits were obtained from him for the described study. The castle and its interior are registered as a monument at the Bavarian State of Office for Monument Protection (monument no. D-5-6729-0021). The conducted analyses were in accordance with the legislative guidelines of the Bavarian Law for the Protection and Preservation of Monuments (BayRS 2242-1-K).

### Coffins

All coffins were photographed, measured and described. Their general shape and fittings were dated by using typo-chronological comparisons to coffins from Northern and Central Germany [23] as well as from Bavaria [24, 25]. The filling of the coffin, such as padding and fabrics, was investigated macroscopically.

### Human remains

#### Physical anthropological investigation

Basic anthropological data were recorded on site using primary and secondary sexual characteristics and robusticity for sex estimation. The dentition was used for a rough estimation of age at death.

**Fig 4:**
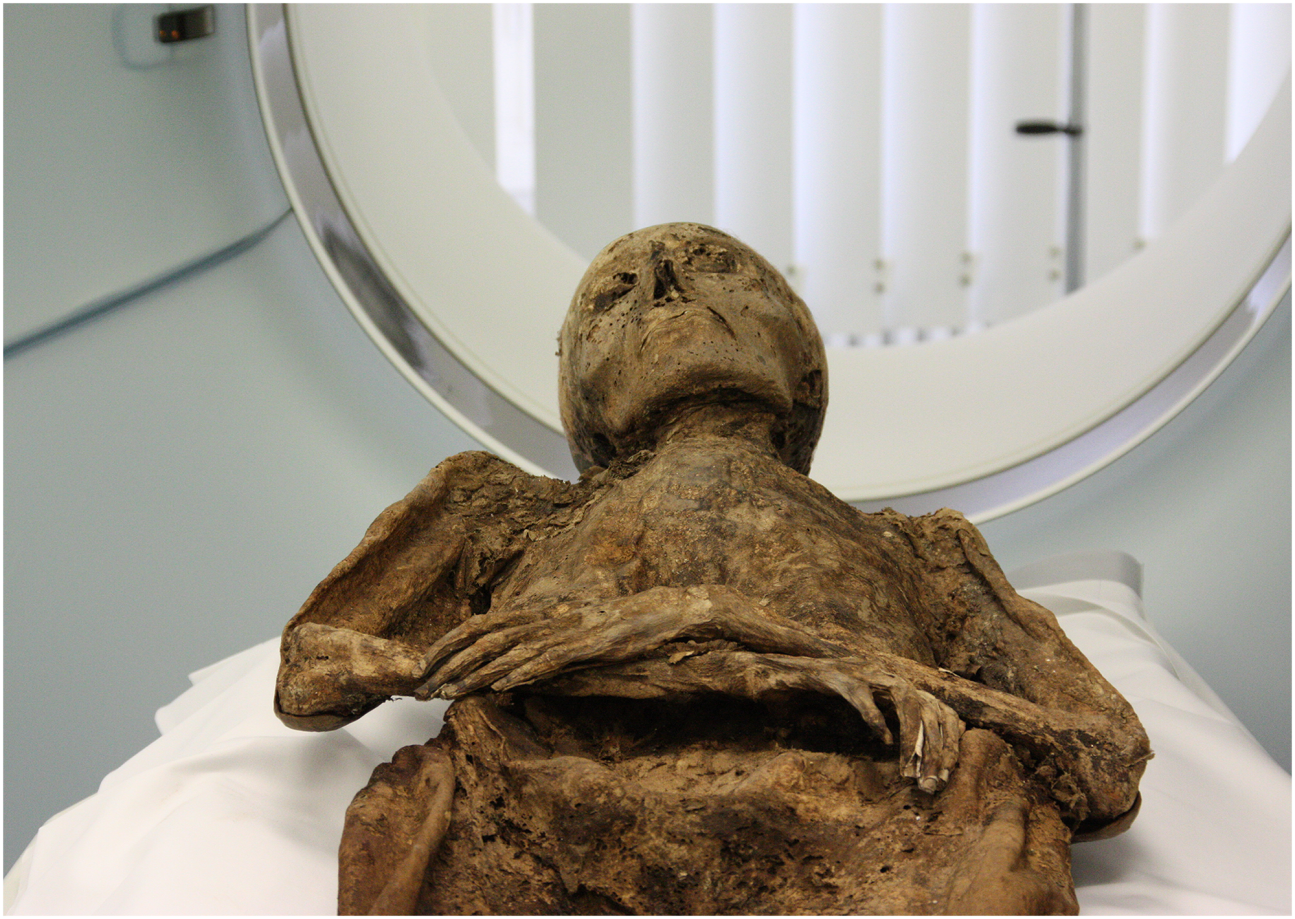
Individual A during CT scanning at Institute of Clinical Radiology and Nuclear Medicine of the University Medical Center Mannheim, Germany (photo: W. Rosendahl).

Following conservation to ensure stability of the mummies through a supporting shell [26], computed tomography was performed on several occasions. Individual B was scanned using a Siemens Somatom Sensation 16 (Siemens Healthcare GmbH, Erlangen, Germany) at the Department of Radiology at St. Theresa’s Hospital in Mannheim (Germany). The individuals A, C, D and G were scanned at the Institute of Clinical Radiology and Nuclear Medicine of the University Medical Center Mannheim using a Siemens Definition Dual Source Energy Scanner (Siemens Healthcare GmbH, Erlangen, Germany) (Fig 4). The applied parameters varied from a slice thickness of 0.6 to 5 mm with diverging slice increment and energy levels of 90 to 150 kV. Due to advances in CT scanning units and software, scan quality was better for the later scanned individuals D and G. Post-processing and evaluation of the DICOM data was performed using the medical imaging software OsiriX (v.3.8.1, Pixmeo SARL, Geneva, Switzerland), Sectra PACS (v.17.16.3569/2015, Sectra AB, Linköping, Sweden) and a Leonardo workstation (Syngo Somaris/7, Siemens Healthcare GmbH, Erlangen, Germany).

CT imaging was used in this research to assess a variety of parameters, including bone and soft tissue preservation [27], post-mortem damage [28], type of mummification [10, 29], age at death, sex, pathologies, trauma, and cause of death [30].

Sex was re-assessed on the digital body using standard anthropological methods [31–33]. In most cases, the presence of genitalia was indicative but skeletal parameters such as supra-orbital ridges and the mastoid process on the skull as well as the greater sciatic notch and the sub-pubic angle on the pelvis were also taken into account. Age estimation in mummies was performed similarly to age estimation of skeletons, even though visibility of the traits is strongly influenced by the quality of the scan data [34, 35]. For this reason, age was estimated on both directly visible (e.g., teeth through the open mouth, cranial sutures through soft tissue damage) and digitally evaluable criteria. The digital age characteristics were the following: fusion of epiphyses [32], general state of dentition, including dental wear and ante-mortem tooth loss [36, 37], closure of the cranial sutures [31, 38], condition of the joints and the spine [39], ossification of the thyroid cartilage [40, 41] and changes to the pubic symphysis and auricular surface [31, 42, 43]. The final outcome is a synoptic comparison of all evaluable age indicators.

The age of the infant mummy was estimated through bone length and skeletal maturation [44–47]. As the head of this mummy was not available, skeletal maturation could not be compared to dental development.

Body height was calculated based on the maximum length of the right femur (F1) after Martin [48] by using the regression formula of Breitinger [49] for males and Bach [50] for females. The regression equations are:

Body height (H) = 94.31 + 1.645*F1 ± 4.8 cm for males [49]
Body height (H) = 106.69 + 1.313*F1 ± 4.1 for females [50].

Femoral length was either measured on the dry bone or on the CT images [51]. Infant body height was determined according to the tables of Schmid and Künle [46].

The state of dentition was used as an age indicator but also to evaluate dental health and nutritional habits [52, 53]. As the recording relied on digital CT images with varying quality and on-site observations, the following parameters were selected to assess the dental status:

1. Number of present teeth, including root residues
2. Number of root residues
3. Teeth lost *ante-mortem*
4. Carious teeth, including root residues
5. Destructive periodontal alterations (e.g., abscess).

Carious lesions could only be observed if they had reached a certain size (*caries media*). It was not possible to record dental wear and enamel hypoplasia due to the quality of available CT images.

Abnormal alterations of bones and soft tissue were described, evaluated and diagnosed [54, 55].

#### Ancient DNA analysis

Ancient DNA (aDNA) analysis was conducted to investigate a possible maternal relationship between the mummies. The analysis was carried out on the hypervariable regions of the mitochondrial DNA (mtDNA) [56, 57]. It was tested whether the individuals A, C, D, E and G share the same sequence in the hypervariable region, possibly indicating a maternal relationship. As only one of the probed individuals was male, Y-chromosomal targets were not used.

The analyses were performed in the clean laboratories of the Centre for Evolutionary Medicine, Institute of Anatomy at the University of Zurich and the Institute for Mummy Studies at the Eurac Research in Bolzano, which are especially dedicated to the work with ancient DNA. Five DNA samples were analysed in the aDNA laboratory at the University of Zurich (Table 1). Since PCR analysis is highly prone for external contamination, the analysis was replicated for two individuals in the aDNA laboratory of the Eurac Research in Bolzano. Rib samples were newly taken for this additional analysis which derived from the interior of the individuals. DNA was extracted, amplified, cloned and sequenced over the mitochondrial hypervariable regions I and II (HVR-I, HVR-II) using overlapping PCR fragments of different sizes. The detailed protocol for both laboratories is given in the supporting information S2 File.

Six mtDNA sequences were aligned to haplogroups (HG) using online mtDNAmanager (http://mtmanager.yonsei.ac.kr) [58].

### Historical records

The available historical records were used to create a list of individuals probably entombed in the crypt (Table 2). Information like name, occupation, ancestry, marital status, date of birth and death and circumstances of death were extracted from the sources [13, 59]. In most cases, the death register is indicating a specific place of interment.

**Table 2:** Individuals entombed in the crypt of Sommersdorf castle according to archival records. Discrepancies between the biographical data and the age might be due to inconsistencies in different sources.

Name	Née	Date of Birth	Date of Death	Date of Interment	Age (death register)	Circumstances of Death	Place of Interment
"Freiherr von Holz" (Baron of Holz)						Thirty Years`War?, according to Bergmann 1833	
Sophia Louise von Kniestätt	von Crailsheim	February 28, 1649	September 5, 1690	September 7, 1690	42 years 6 months 5 days	Death in childbed, seizures	
Agatha Magdalena von Crailsheim	von Hüffel	1659	June 8, 1713	July 21, 1713 / December 1717	54 years 15 days		Transfer of coffin into crypt in Sommersdorf (next to her husband)
Georg Wolff von Crailsheim		April 24, 1655	December 13, 1717		63 years 7 months 2 weeks 1 day	Apoplexy	Crypt in Sommersdorf (next to his wife)
Helena Friderica von Rauber	von Crailsheim			November 2, 1760	66 years		Baronial crypt in Sommersdorf
Ernst Heinrich Gabriel von Soden		August 1, 1713	August 17, 1761	August 20, 1761	48 years	8 days lasting sickness	Baronial crypt in Sommersdorf
Wilhelmine Charlotta von Soden	von Rauber	February 12, 1721	March 3, 1766	March 26, 1766	46 years		Baronial crypt in Sommersdorf (next to her mother and husband)
Karolina Elisabetha Christiana Charlotta von Crailsheim		December 17, 1769	April 6, 1770	April 8, 1770	4 months	Catarrh, convulsions	Crypt in Sommersdorf
Friedrich Franz Wilhelm von Crailsheim		January 22, 1771	January 29, 1771	January 31, 1771	8 days	Convulsions	Crypt in Sommersdorf
Friedrich Karl Wilhelm von Crailsheim		June 10, 1772	June 18, 1772	June 20, 1772	9 days	Convulsions	Crypt in Sommersdorf
Charlotta Eleonora Karolina von Crailsheim		April 15, 1774	August 31, 1775	September 2, 1775	1 year 4 months	Convulsions	Crypt in Sommersdorf
Eleonora Christiana Ernestina von Holz	Schenck von Geyern	September 27, 1725	March 19, 1783	March 21, 1783	58 years		Baronial crypt in Sommersdorf
Julius Wilhelm von Crailsheim		September 30, 1764	March 10, 1812	March 12, 1812	47 years 5 months 9 days	Rheumatic seizures & meningitis vs. hunting accident	Crypt in Sommersdorf

## Results

### Coffins

All coffins are made from oak and consist of a single outer coffin. They have a trapezoidal shape and either straight or fluted side plates (Fig 1). The coffins are mostly undecorated with the exception of a wooden cross on some lids. The handles are quite uniform with rhomboid-shaped fittings in floral-ornamental style and multi-noded grips. They can be dated to the late 17^th^ to early 19^th^ century AD. Due to their fluted side plates, the four children’s coffins no. 6, 8, 9, 10 can be dated more precisely to the second half of the 18^th^ century while coffin no. 4 can be attributed to the late 18^th^ or early 19^th^ century.

At that time the coffins were individually manufactured for the deceased, and for this reason their length was adapted to the bodies’ height. The children’s coffins measure between 72 and 100 cm in length while the adults’ coffins range from 158 to 202 cm. It is evident that coffin lid no. 7 which has a length of 158 cm would be too short to contain the body of a fully grown person, and several indicators point towards a re-working of this lid. This re-working probably took place after one of the numerous openings and restorations of the crypt in the years 1806, 1822, 1864 or 1871 [13].

The coffins are filled with wood shavings and lined with fabrics. During restoration, it appeared that both were probably renewed at the end of the 19^th^ or beginning of the 20^th^ century. However, some of the original wood shavings are stuck to the mummies’ skin.

### Mummies

#### Physical anthropological investigation

Bone and soft tissue preservation of the mummies is quite good. The overall representation of the mummies is adequate for anthropological investigation and anatomical integrity of the mummies is maintained even though the individuals E, F and G are incomplete. Several articular dislocations and soft tissue damage attest the multiple violations of the crypt.

Skin, muscles, tendons and bones can be distinguished in the CT images. Soft tissue preservation was further evaluated for the scanned individuals A, B, C and D: Inside the skulls, remnants of the shrunken brain and the meningeal membranes are observed. In the thoracic cavities remnants of the lungs and the mediastinal structures are found in all cases, including the heart, the trachea and the aorta.

In the abdominal cavities only minor remnants of anatomical structures could be observed due to their shrinkage and decomposition.

**Fig 5:**
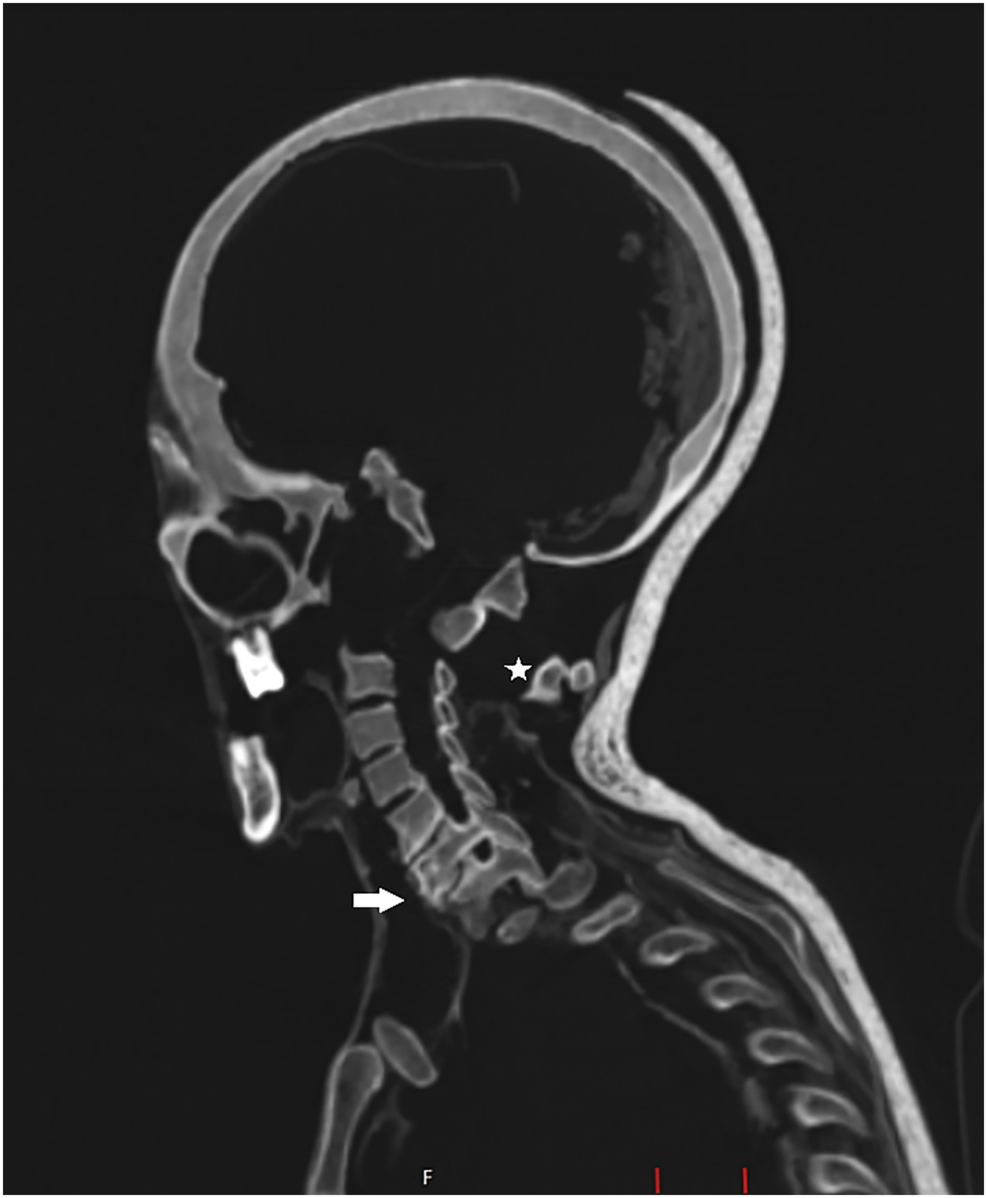
Sagittal multi-planar reconstruction of a CT data set showing the skull and the cervical spine of individual A. Remnants of the shrunken brain are visible in the posterior cranial fossa. The lower cervical and upper thoracic spine reveals degenerative changes (white arrow). The cranio-cervical junction depicts severe displacement of the atlas and axis (asterisk).

**Individual A:** The mummy is lying in an extended supine position with the arms bent in a 90-degree angle and resting on the chest. The left hand is lying on the right forearm (Fig 4). Remnants of clothing still cling to the mummy’s skin, and white leather gloves and knitted woollen stockings are preserved (Fig 3). As a result of post-mortem manipulation, the osseous cranio-cervical junction is no longer maintained (Fig 5).

**Fig 6:**
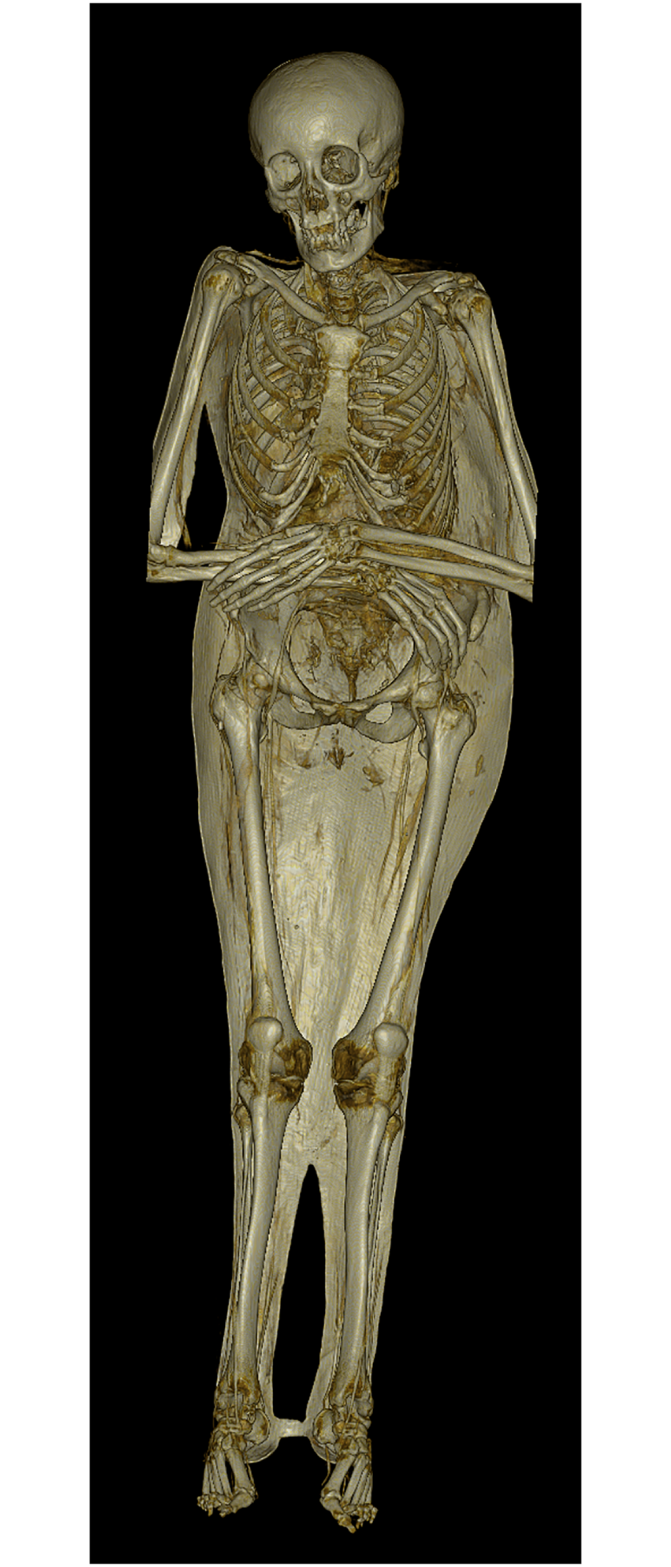
3D volume rendering of a CT data set representing individual A. The mummy is placed on a supporting shell. The dentition shows ante-mortem tooth loss. Note the asymmetry between the torso and the lower extremities due to scoliosis.

**Fig 7:**
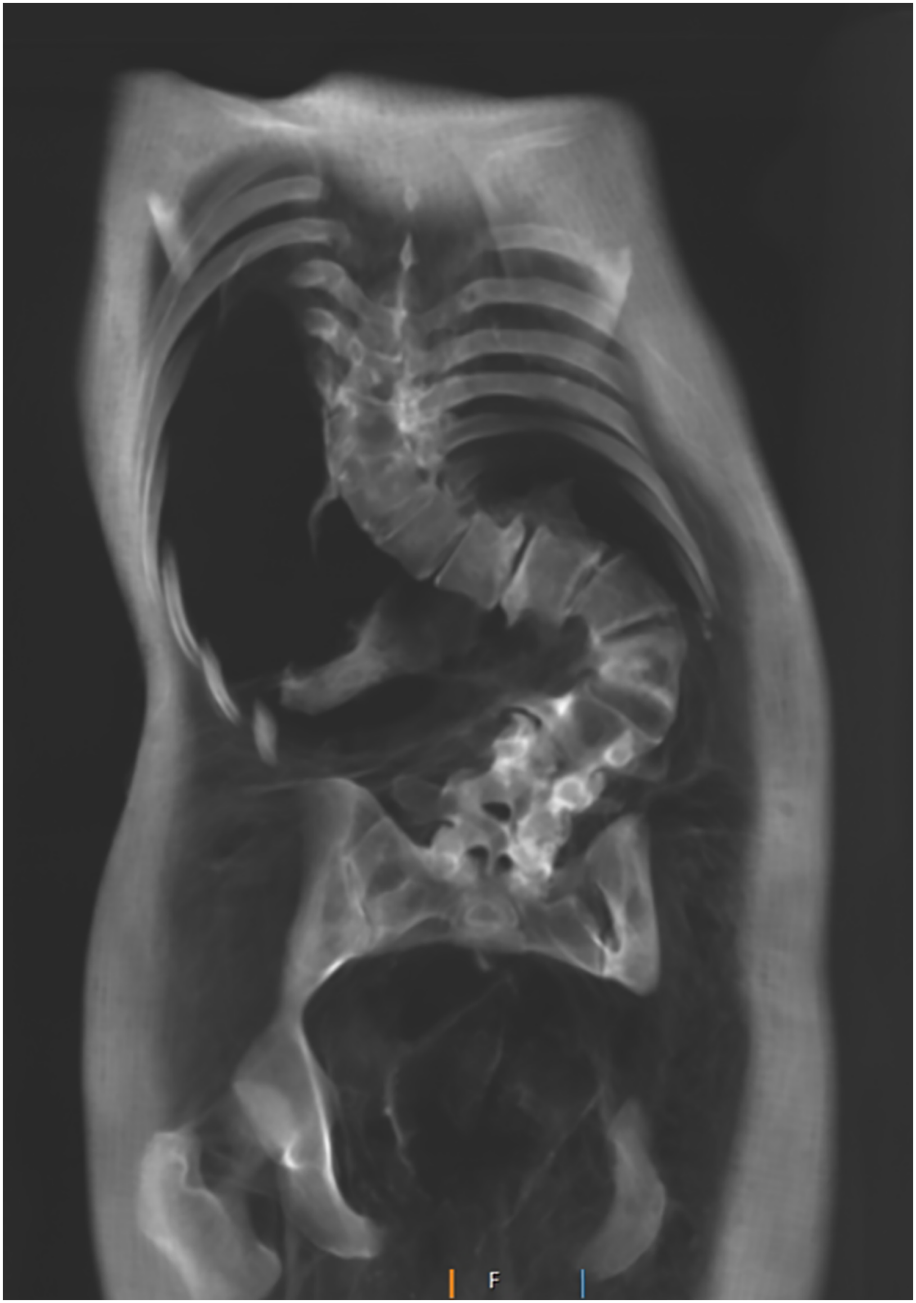
Coronal multi-planar reconstruction of a CT data set depicting the spine of individual A with severe scoliosis and secondary degenerative changes.

The individual is female, age 40 to 60 years, and shows a supernumerary non-sacralized 6^th^ lumbar vertebra and a perforated xiphoid process as anatomical variants. On the CT images, a lateral deviation of the spine was recognized in the lower thoracic and lumbar vertebrae (Figs 6 and 7). The lumbar vertebrae are bent towards the left side of the body. The vertebral bodies of L2 and L3 exhibit lateral wedging at the apex of the curvature in addition to rotation and torsion. Since the curvature continues over several spinal segments and no primary narrowing of the intervertebral space could be observed (as it would be expected in tuberculosis [60]), the curvature is most likely caused by an extreme left-convex lumbar scoliosis with a right-convex counter-curve. Furthermore, resolution of cancellous bone tissue could be observed in the vertebral bodies of L2 and L3. The lesions are sharply defined and limited to the bodies without affection of the roof plates. A possible diagnosis could be an intraosseous vertebral haemangioma. Haemangiomas are benign, solitary tumours of proliferating blood vessels and vascular sinusoids that are mostly asymptomatic and usual incidental findings of imaging techniques. Additional bone changes of the spine include extreme degenerative alterations to the superior and inferior articular facets as well as to the bodies of the lower thoracic and lumbar vertebrae. It is probable that mobility was reduced due to this deformity but complete ankylosis of the vertebral bodies L1 to L5 is not present [18]. In addition, slight arthrosis was found in the left sacroiliac joint. The cervical vertebrae C6 and C7 as well as the first thoracic vertebra T1 exhibit localised degenerative changes to the vertebral bodies, including sclerotic rims on the vertebral plates (Fig 5). Those alterations can be interpreted as signs of advanced cervical and upper thoracic osteochondrosis. The individual further displays a bone cyst in the left distal ulna. Body height is estimated to have been 163.1 cm.

Based on the family chronicle [13], individual A was usually identified as *Baroness Schenck von Geyern*.

**Fig 8:**
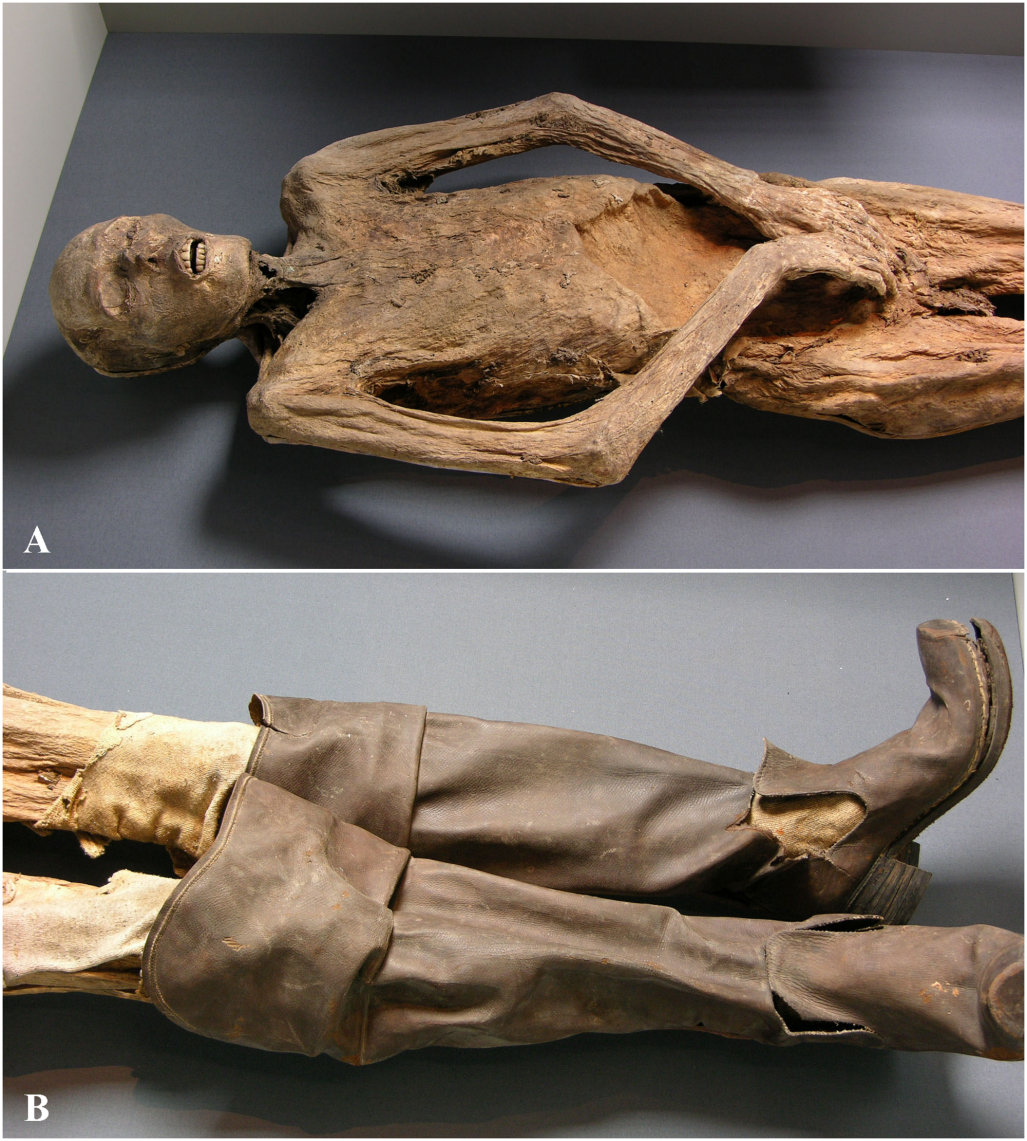
Individual B. (A) during the exhibition “Mummies—The Dream of Eternal Life” in Mannheim, Germany, 2010 (photo: W. Rosendahl). (B) Preserved leather boots. Characteristic features are the square domed toe, the high stacked heel and the openings to the sides (photo: W. Rosendahl).

**Individual B:** The mummy is lying in an extended supine position with the hands placed next to each other on the lap (Fig 8A). The mummy is still dressed with gloves, knee-high leather boots and woollen knitted stockings underneath (Fig 8B). Post-mortem damage affects the anterior portion of the neck.

**Fig 9:**
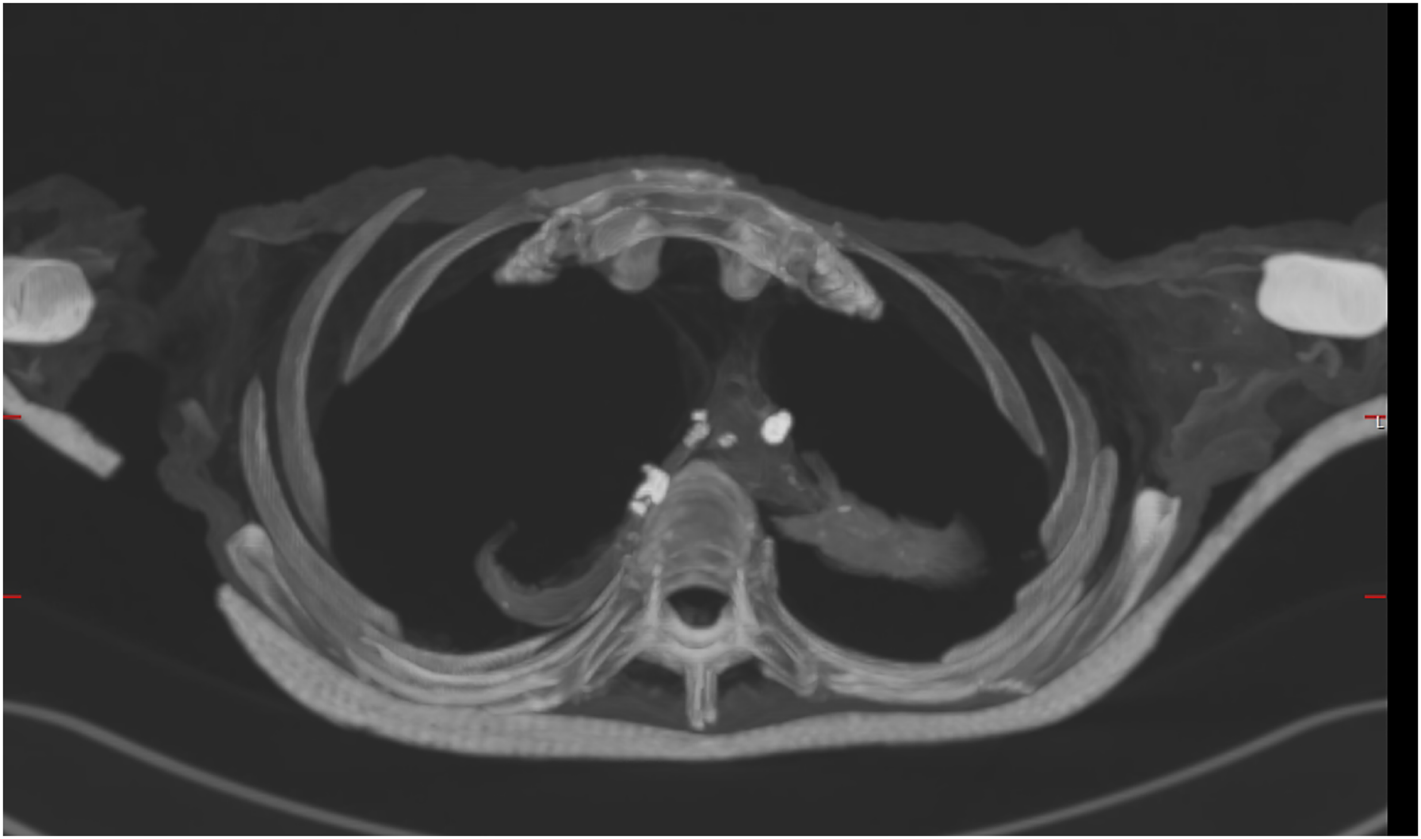
Axial multi-planar MIP reconstruction of a CT data set showing the thorax of individual B. Several calcifications can be seen in the mediastinum, the pulmonary hili and the lungs.

The individual is male, aged 25 to 40 years, and displays several anatomical variants, among them a non-sacralised 6^th^ lumbar vertebra and a frontal sinus aplasia. No degeneration of the spine or the joints was observed. Several smaller calcifications are located in the thoracic as well as abdominal parts of the aorta, the hili of the lungs and in the trachea (Fig 9). Another structure of ovular shape, approx. 1 cm in diameter and a radiodensity of 1600–2000 Hounsfield Unit (HU) is found within the left paramediastinum. Shell-like radiodense structures of different size in the right thoracic cavity and abdomen might represent the content of the intestines. The estimated body height is 172.8 cm.

Individual B was commonly designated as *Baron von Holz* [13].

**Fig 10:**
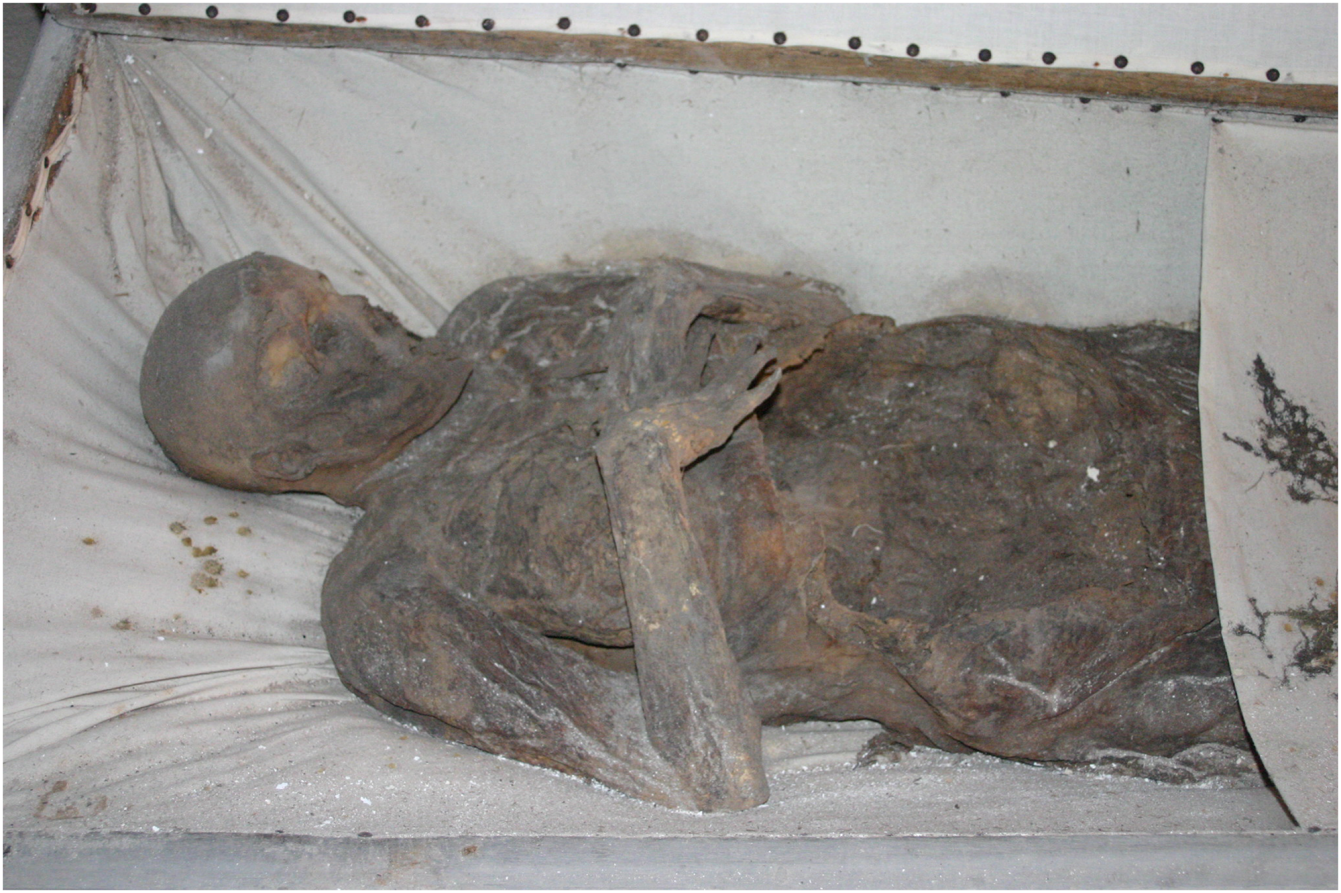
Individual C in her coffin in Sommersdorf. The coffin is lined with fabric. Post-mortem damage can be seen in the facial area and at the abdomen (photo: W. Rosendahl).

**Individual C** was buried in an extended supine position. The arms are bent in a 50 degree angle so that the hands—probably formerly interlaced—have sunken on the breasts (Fig 10). Individual C shows severe post-mortem damage and lacks the complete anterior abdominal wall as well as soft tissue over the sacrum, the upper spine and the upper facial area. Additionally, the individual has several dorso-lateral rib fractures on the left side indicating a rough manipulation of the mummy in the past.

**Fig 11:**
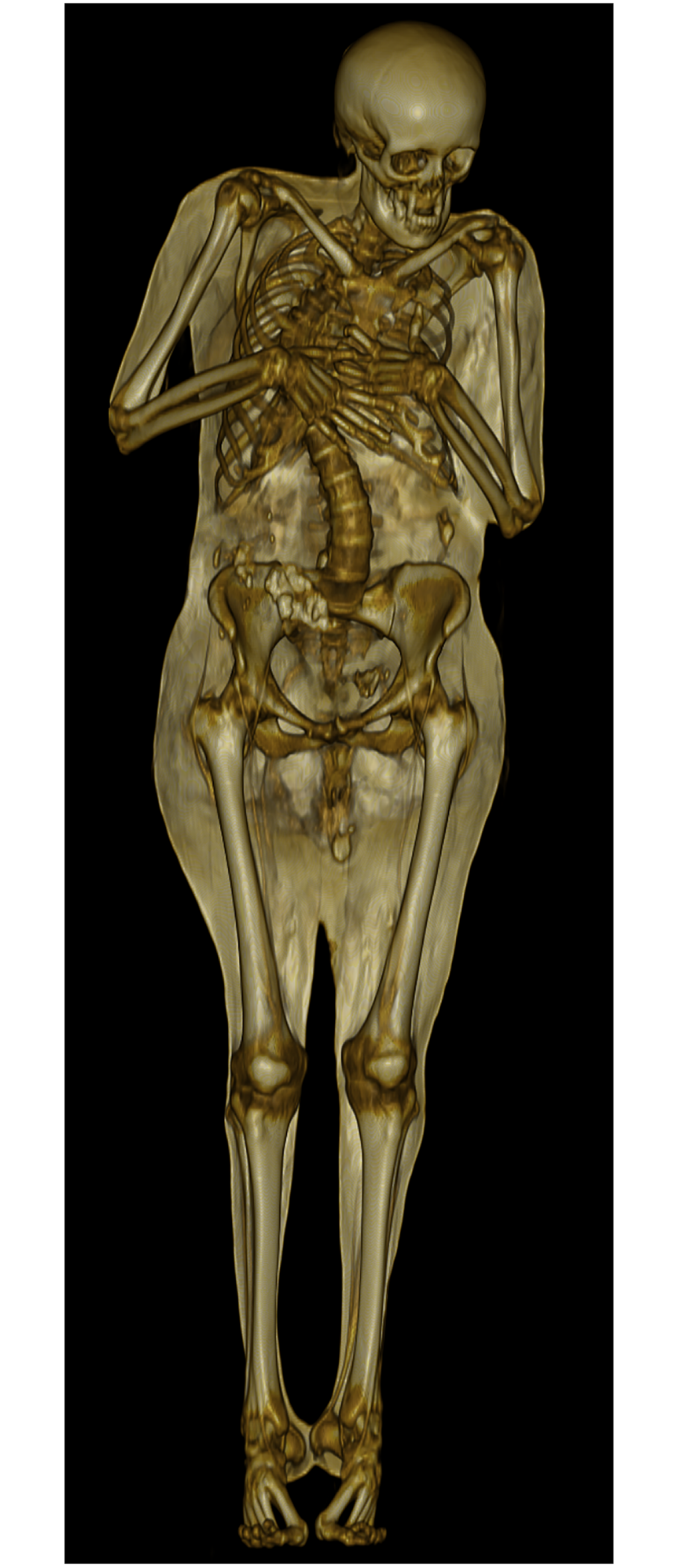
3D volume rendering of a CT data set representing individual C. The mummy is placed on a supporting shell. The dentition shows ante-mortem tooth loss. The thoracic vertebrae exhibit lateral deviation (scoliosis).

**Fig 12:**
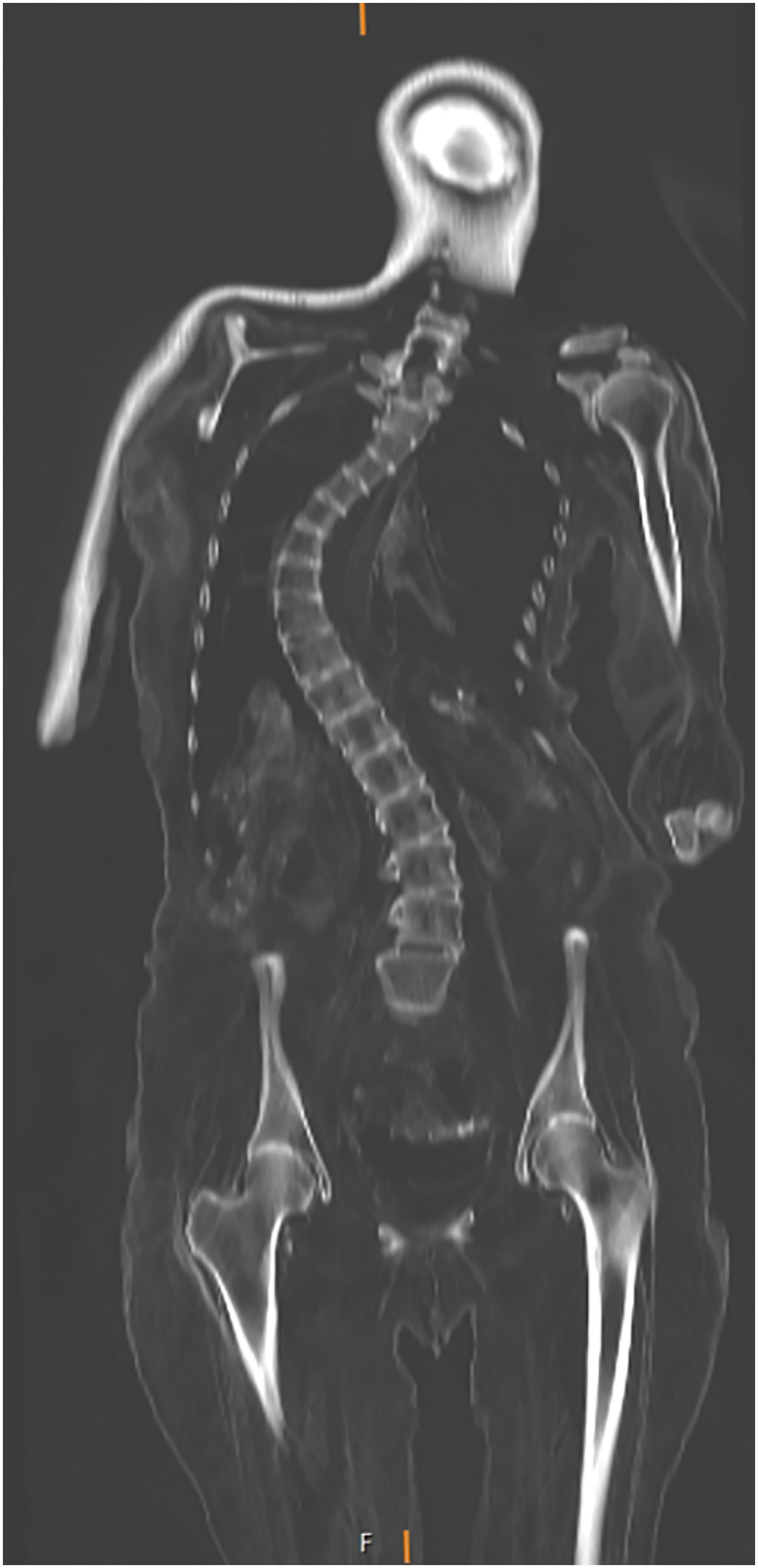
Coronal multi-planar reconstruction of a CT data set showing the spine of individual C with lateral deviation (scoliosis).

**Fig 13:**
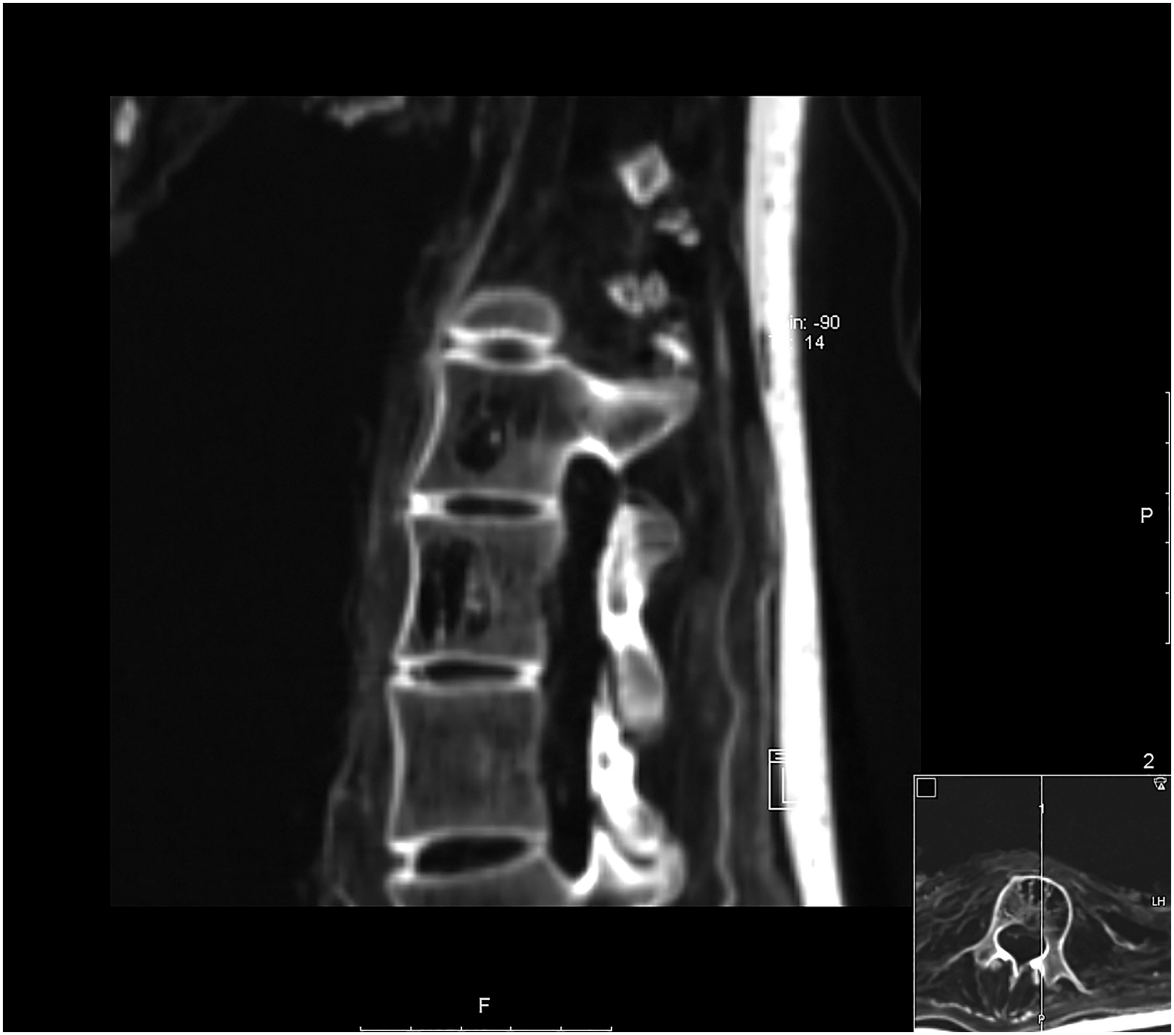
Sagittal multi-planar reconstruction of a CT data set showing the lumbar spine of individual C. In the sagittal view, the lumbar vertebrae 1 and 2 show sharply confined lesions of honeycomb appearance, also visible in the axial view (small window). The lesions were probably caused by intraosseous vertebral haemangiomas.

The individual is female, aged 30 to 50 years. A lateral deviation of the spine was observed on the CT images (Fig 11). It can be described as right-convex, thoracolumbar scoliosis with the apex at the vertebra T8 (Fig 12). Degenerative alterations are present on the articular facets of the lower thoracic and lumbar spine. The vertebral bodies of L1 and L2 exhibit lesions in the cancellous bone (Fig 13). In sagittal view, the lesions have a sharply confined and of locular form. In axial view, the honeycomb appearance is also observable. The roof plates of L1 and L2 are intact and the intervertebral space does not exhibit any narrowing which would exclude tuberculosis as a differential diagnosis [60]. The lesions were probably caused by an intraosseous haemangioma [54]. The pubic symphysis shows alterations in form of a joint irregularity and sclerotic rims. Both might be signs of recurrent stress, advanced age and/or a preceding inflammation of the symphysis. The left sacroiliac joint shows similar alterations with joint surface irregularity and sclerotic rims. Both sides exhibit a misalignment with a protruding sacrum.

Besides, individual C exhibits particularities that are probably related to overweight: the mummy’s breasts and thighs are pronounced, and a fat apron is found on the buttocks. The estimated body height of individual C is 164.5 cm.

Individual C is popularly known as *Sophie Luise von Kniestätt* [15].

**Individual D** was buried in an extended supine position with the hands placed on top of each other on the abdomen (Fig 3). The individual is wearing woollen knitted stockings with a geometric pattern. The soft tissue on the anterior neck and the facial area shows heavy post-mortem damage, possibly aggravated by decomposition. Some reddish curly hair is still preserved on the head.

**Fig 14:**
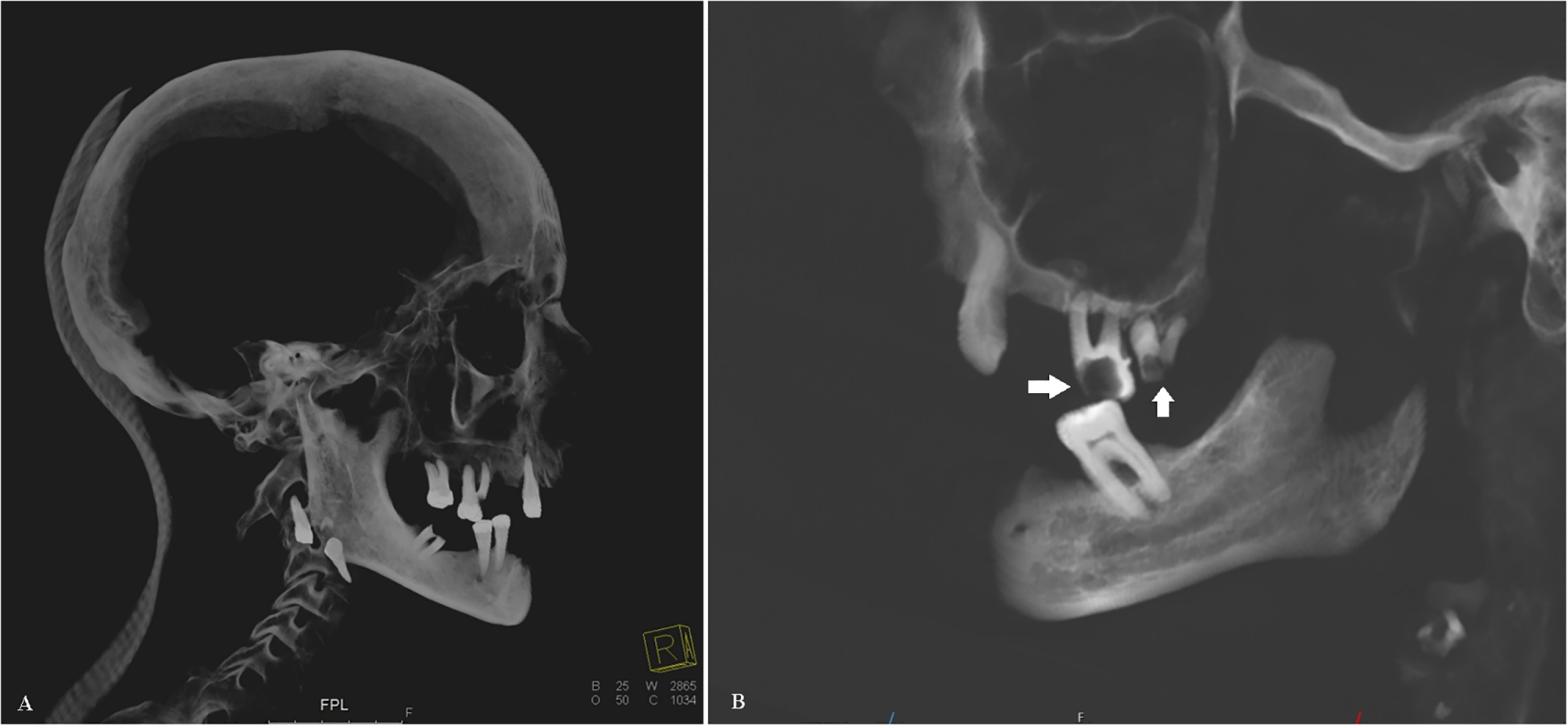
Sagittal multi-planar MIP reconstruction of a CT representing the skull of individual D. (A) The dentition shows several alterations including ante-mortem tooth loss, carious lesions, destructive periodontal processes, and post-mortem displacement of teeth. (B) Detail of the dentition showing ante-mortem tooth loss and severe carious lesions (arrows).

Individual D is male with an estimated age of 35 to 55 years. He exhibits several ante-mortem and post-mortem alterations of the dentition (Fig 14A and 14B), including a dislocation of several teeth in the oral cavity and the upper half of the cervical part of the trachea. His fifth lumbar vertebra shows an additional articular facet towards the sacrum on the right side. Schmorl’s nodes are present in the thoracic vertebrae. The calculated body height is 169.7 cm.

Individual D is believed to be *Julius Wilhelm von Crailsheim* [13].

**Individual E** is lying in an extended supine position on the bare bottom of the coffin. The arms are bent and lying parallel to each other on the chest. The mummy was damaged at the neck, the chest and feet, resulting in the absence of the mandible and the feet. No remnants of clothes were found.

Individual E is female with an estimated age of 30 to 50 years according to the state of dentition. No imaging data are available for this individual. The estimated body height is 160.1 cm.

No identity has been ascribed to individual E.

**Individual F** is found in the reversed coffin lid no. 7. It is partially mummified without head, left arm, right forearm and lower legs. The original body position cannot be determined. The individual is male and 40 to 60 years of age. The individual has osteophytes on the spinous processes of the lower thoracic and lumbar vertebrae as well as enthesophytes on the pelvis and sacrum. The latter are especially pronounced on the iliac crest and the ischial tuberosity and may be related to old age, obesity, or repeated acute minor stress due to physical activity. They can also occur in diffuse idiopathic skeletal hyperostosis (DISH) [61]. Further differential diagnosis was hampered by the integrity of the abdomen and chest and the lack of imaging data. The calculated body height is 165.5 cm. Individual F does not have an ascribed identity.

**Fig 15:**
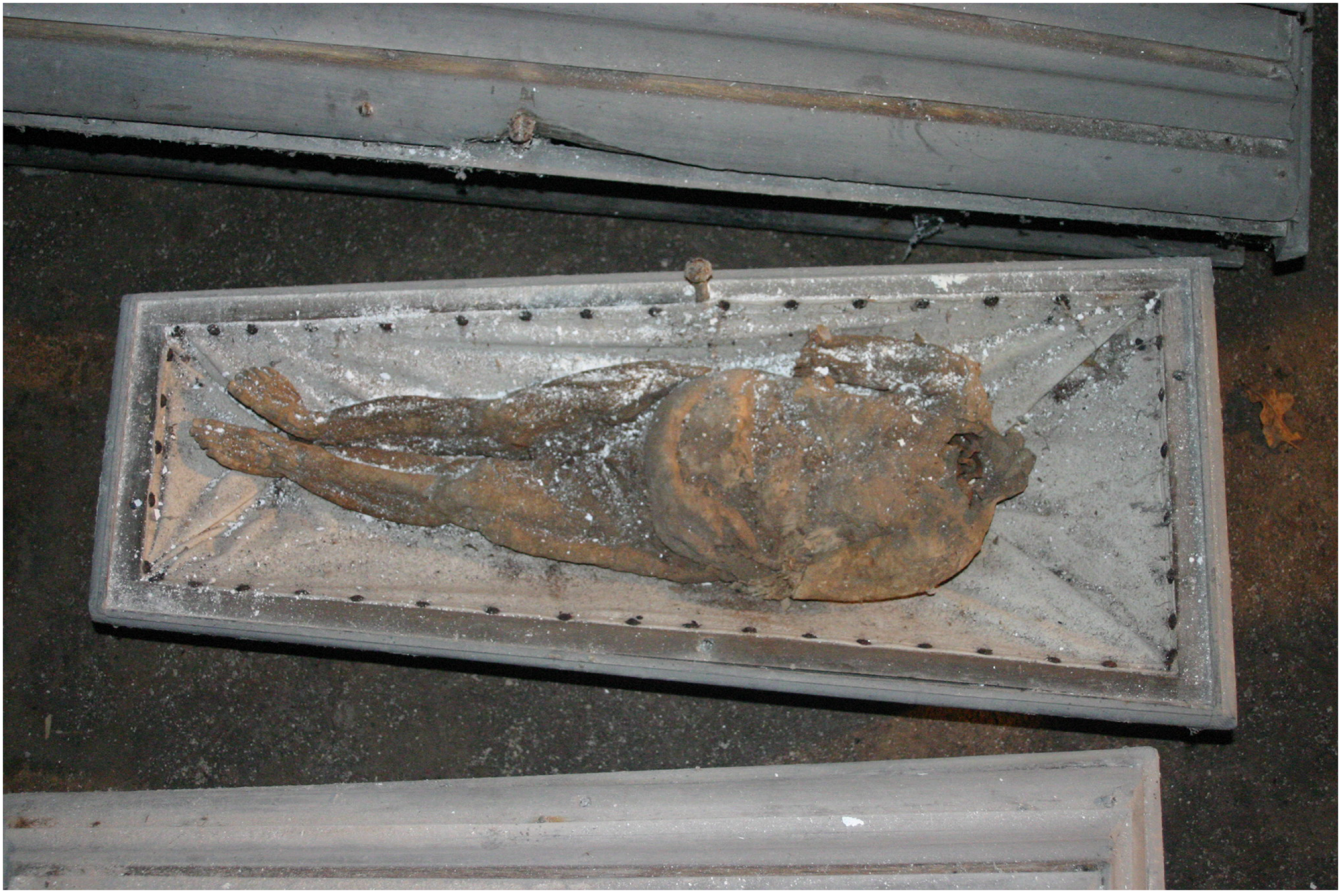
Infant mummy individual G in its coffin. The coffin is lined with fabric. The mummy’s head and forearms are missing (photo: W. Rosendahl).

**Fig 16:**
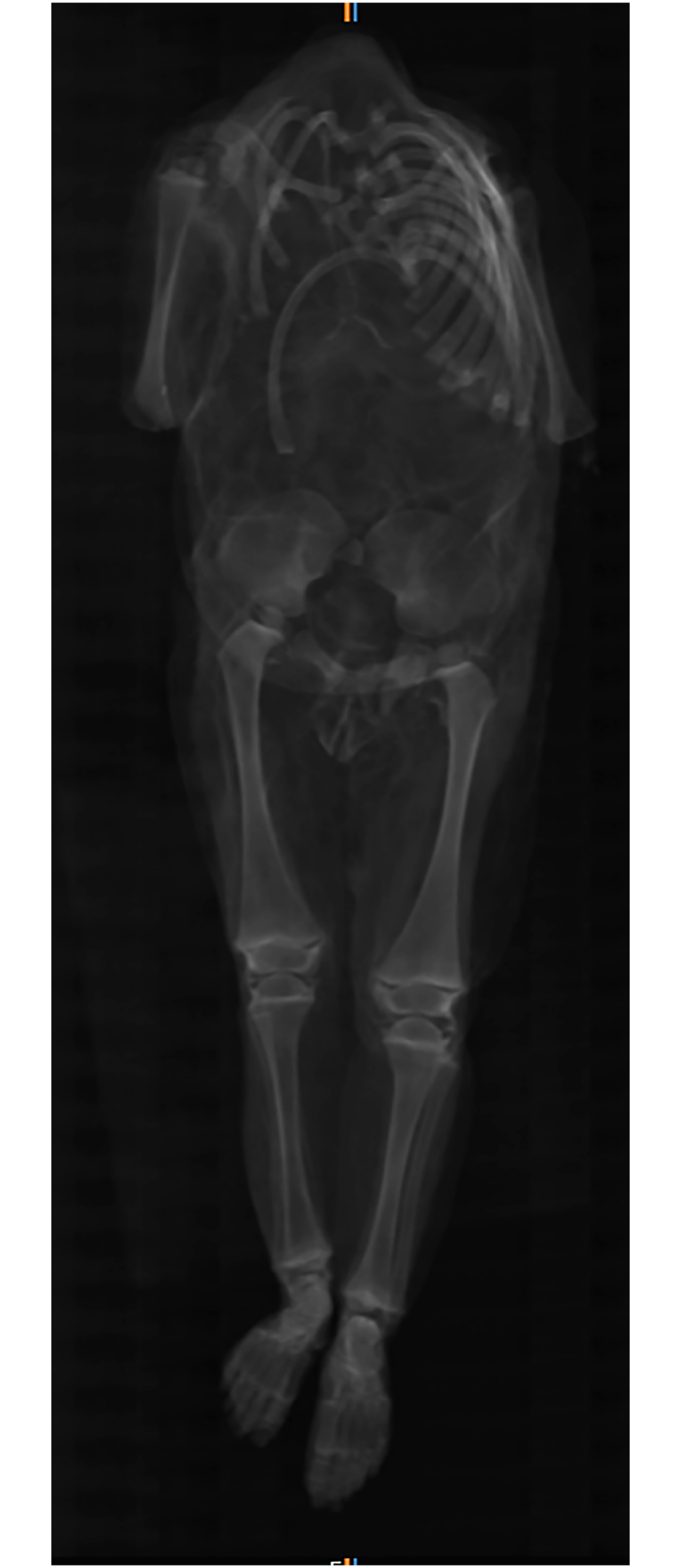
Coronal multi-planar reconstruction of a CT data set showing individual G. The trunk skeleton is largely disintegrated. Note the presence of the ossification centres of the proximal humeral head, proximal femoral head and distal femur, proximal and distal tibia, and distal fibula.

**Individual G** is an incomplete infant mummy without head or forearms (Fig 15). The elements of the trunk skeleton are completely disintegrated due to post-mortem damage (Fig 16).

The infant mummy could be identified as female due to observable genitalia and was aged 6 to 12 months old according to long bone length. Ossification centres of the following epiphyses are present: proximal humeral head, proximal femoral head and distal femur, proximal and distal tibia, distal fibula, calcaneus, talus, cuboid and lateral cuneiform (Fig 16). Therefore, the infant can be estimated older than 3 months. However, the ossification centres of the proximal fibula, the medial and intermediate cuneiform as well as the navicular are absent which allows to narrow down the age to below 8 months [47]. The calculated body height is 67 cm.

No remnants of organs could be identified in the thorax or abdomen. No identity has been ascribed to individual G.

**Individual H** was entombed in extended supine position with the arms bent in a 90 degree angle and lying parallel to each other on the chest (Fig 3). The right hand is placed on the left forearm. The individual is wearing knee-high woollen knitted socks and leather gloves.

Individual H is a 25 to 45 year old female. The estimated body height is 163.8 cm. No imaging data are available for this mummy.

Individual H is believed to be the wife of *Major von Soden*.

Table 3 summarizes the results of the anthropological investigation. In total, four of the seven adult mummies are females and three are males. The scattered elements found in the coffins no. 5, 6 and 10 represent a left mummified forearm including the hand, as well as a left humerus, right radius, left radius, right ulna, left ulna, some hand bones, right femur, left femur, right tibia, left tibia, and a left fibula. They can be attributed to a minimum number of two adult individuals. One individual represented by two femora of the same length and shape is *probably female* with an estimated body height of 165.6 cm. The left mummified forearm could belong to individual F due to its length and robusticity. Individual G represents the only immature individual in the crypt. In total, a minimum number of nine individuals were found in the crypt (Table 3).

**Table 3:** Summary of the anthropological results.

Coffin no.	Ind.	Sex	Age (in years)	Length (right F1) (cm)	Calculated body height (cm)	Dentition (parameters a-e)	Anatomical variants	Pathologies and possible diagnoses
**1**	**A**	female	40–60	43	163.1	a) 18	6^th^ lumbar vertebra, perforated xiphoid process	Scoliosis, haemangioma, bone cyst
						b) 2		
						c) 14		
						d) 3		
						e) 2		
**2**	**B**	male	25–40	47.7	172.8	a) 32	6^th^ lumbar vertebra, frontal sinus aplasia, perforated xiphoid process	Calcifications in the lung and aorta
						b) 0		
						c) 0		
						d) 1		
						e) 2		
**3**	**C**	female	30–50	44	164.5	a) 19	/	Scoliosis, haemangioma
						b) 1		
						c) 11		
						d) 3		
						e) 1		
**4**	**D**	male	35–55	45.8	169.7	a) 13	Assimilation of L5	/
						b) 2		
						c) 10		
						d) 5		
						e) 1		
**5**	**E**	female	30–50	40.7	160.1	a) 8*	/	Moderate dental wear
						b) 0		
						c) 2		
						d) /		
						e) /		
**6**		probably female	40–60	44.9	165.6	/	/	Arthrosis on the right patella
**7**	**F**	male	40–60	43.3	165.5	/	/	Degenerative spine changes
**9**	**G**	female	0.25–1	12.2	67	/	/	/
**10**		probably female	40–60	/	/	/	/	Arthrosis on the left patella
**11**	**H**	female	25–45	~43.5	163.8	Not observable	/	/

no. = number; ind. = individual; F1: maximum femur length;

* = only upper jaw

The average body height is 163.4 +/- 4.1 cm for females (n = 5) and 169.3 +/- 4.8 cm for males (n = 3).

In five individuals a total of 77 teeth were observed of which a minimum of 12 teeth showed alterations due to caries (15.6%). This number includes the teeth with carious lesions and the root residues which likely showed alterations due to caries. The percentage of ante mortem tooth loss (27.2%) is based on the observable tooth locations (37/136). If the *ante-mortem* tooth loss is included, the caries intensity is 36.0% [62]. The caries frequency is described as the number of individuals with caries or *ante-mortem* tooth loss in relation to the total number of observable individuals and is 100% in this sample [62].

#### Genetics

Table 4 summarizes the results of the mtDNA analysis. Sequences from hypervariable region I and II (HVR-I, HVR-II) are presented as variant nucleotide positions to the revised Cambridge Reference Sequence. Three different haplotype sequences were found among the investigated individuals (H, U5a1, H2a3). Three individuals showed the haplotype H, the most common haplogroup in Europe [63]. But even the three individuals of haplogroup H are of different subtypes, as shown by the differences in the nucleotide substitutions (Table 4).

**Table 4:** Mitochondrial DNA haplotype sequences of typed individuals.

Ind.	HVR-I (np 16024–16569)	HVR-II (np 001–437)	Expected HG	Estimated HG
**A**	16192T	150T	H	H
**C**	-	263G 309.1C 315.1C	H	H
**D**	16129A 16192T 16256T 16270T 16399G	-	U5a	U5a1
**E**	-	263G 315.1C	H	H
**G**	16069T 16093C 16126C 16261T 16274A 16355T	-	H2a3	H2a3

ind. = individual; HVR = hypervariable region; np = nucleotide position; HG = haplogroup

These results show that the five tested individuals are not maternally related but do not rule out paternal relationships.

## Discussion

Initially, archaeological and historical records, anthropological data and mtDNA results were assessed independently in order to avoid biased interpretation. However, it soon became clear that the results of each discipline can only be explained in light of the other available information. The following comparative interpretation of the results allows evaluating the strengths, weaknesses and limitations of each method.

### Historical context

The crypt was installed in the parapets, thus in the defence system of the castle. An opening for countersinking the coffins into the crypt connected the overlying church with the parapets. Inhumations could only have taken place there when the parapets were no longer in use, probably after the settlement of major European conflicts, such as the Thirty Years’ War (1618–1648) or the League of Augsburg in 1686 [13].

Due to the historical records, a list of individuals presumably entombed in the crypt could be compiled (Table 2). However, death registers are not necessarily precise about the exact place of interment. For instance, the family chronicle lists Sophia Luise von Kniestätt, née von Crailsheim, as to be entombed in the crypt [13] but the death register only states the fact of her entombment in Sommersdorf without giving a precise location. Furthermore, coffins may also have been relocated into and out of the crypt. Agatha Magdalena von Crailsheim (1659–1713) was only placed into a non-specified crypt following her husband’s death in 1717. It remains unclear whether this crypt was the same as the baronial crypt of the later inhumations. As the coffin of Agatha Magdalena von Crailsheim must have still been intact at that time, she was probably not buried in the churchyard, but placed temporarily within the church, a chapel or the castle itself. Besides the church, a smaller chapel that corresponds to the current *Kasperlturm* was in use during the 17^th^ and early 18^th^ century [11, 13]. It is possible that inhumations also took place in the parapets below this building. In the death record of Agatha Magdalena von Crailsheim, another burial place close to the altar underneath an ancient stone is mentioned that may refer to the burial places of former family members.

Surprisingly, only a few people who were entombed in the crypt were actually members of the Crailsheim family. Sommersdorf was only one residence of the family, and due to their functions for the margraves of Brandenburg-Ansbach the family was usually living in Ansbach or in Rügland Castle [12]. Sommersdorf Castle fell out of the family’s property between 1717 and 1747, and no inhumations are expected to have taken place in the crypt during this time period. After 1747, the castle was only used as residence for widowed family members or later-born sons. Helena Friderica von Rauber, née von Crailsheim, and her daughter Wilhelmina Charlotte von Soden (Table 2) are good examples for this practice as they were probably living in Sommersdorf while their husband and father Christoph Wilhelm von Rauber (1685–1763) was imprisoned at the fortress of Wülzburg [64]. Eleonora Christiana Ernestina von Holz, née Schenck von Geyern (1725–1783) and married to Gottfried von Holz (1716–1777), probably moved to Sommersdorf after her husband’s death in 1777 in order to live on her son-in-law’s estate [65]. Working as a patrimonial judge, Julius Wilhelm von Crailsheim (1764–1812) was living in Sommersdorf while his cousins used the residencies in Ansbach and Rügland due to their fathers’ prior rights.

It is important to note that sources other than the Sommersdorf church register independently claim the stay and interment of the above-mentioned individuals in Sommersdorf [19, 65] which mutually confirms their authenticity and accuracy [66].

### Funerary customs

The coffins do not show major differences in manufacture or quality. All are simple wooden coffins without any inscriptions, name plates or metal fittings (Fig 1). Differences in shape derive from the typo-chronological development of the coffins, dating them to the late 17^th^ to early 19^th^ century AD, which corresponds to the entries in the death register. The applied wooden crosses seem to be typical for Franconia as crosses were usually painted on the coffins in white colour in other regions [9, 67]. Even though comparative material from the region is scarce, the coffins might indicate an impoverishment of the Crailsheim family branch in Sommersdorf. This observation would fit to the historical sources that mention Sommersdorf as a secondary family residence [12].

The entombed individuals must have been originally dressed [68]; however, clothes were damaged and stolen by Napoleon’s troops in 1806 and by curious onlookers after the renovation of the crypt in 1822 [14]. Some remains of clothing still adhere to the mummified skins. The individuals A, B, D and H are wearing woollen knitted stockings and individuals A, B and H in addition white leather gloves (Fig 4). The gloves suggest that the individuals were dressed in normal or festive clothes instead of a special burial garment. According to gloves, boots and buttons, individual B might have even been entombed in a uniform. The clothing indicates that the bodies were laid out during the funeral service before they were entombed in the crypt [69]. Only for individuals B and E whose bodies were found without clothing remains a burial garment or shroud might be considered.

If clothes had been better preserved, a more precise dating of the inhumations would have been possible [9]. Individual B is wearing nearly unworn knee-high leather boots with a wide square domed toe, a high stacked heel and openings to the sides that can be dated to the second half of the 17^th^ century (Fig 8b) [70, 71]. On the lithograph of Josef Bergmann from 1833 (Fig 3), this individual is shown with a rapier on his side. The sword is nowadays lost but from the lithograph its form can be attributed to the 17^th^ century. In addition, inhumations with rapiers are generally more typical for the 17^th^ than for later centuries, for example in Crailsheim [72] and Sulzbürg [4].

### Circumstances of mummification

Mummification is the process by which soft tissue decomposition after death is halted or significantly slowed, resulting in long-term preservation. In most forms, soft tissue preservation occurs when tissue dehydration slows or halts postmortem decay. This may be achieved either naturally, as a result of environmental conditions, or by intentional human practices that result in artificial mummification [73].

None of the mummified individuals showed signs of an artificial mummification. No incisions were visible on the trunk, such as described by Mylius [74] and Colleter et al. [75] for Central Europe, and the skulls were intact so that no removal of the brain or other organs has taken place, such as seen by Piombino-Mascali in Sicily [5, 76]. Remnants of the shrunken brain, heart, aorta and trachea were observable on the CT images. The abdominal cavities were largely empty in comparison to the thoracic cavity as a consequence of putrefaction and organs comprising less connective tissue [77]. No foreign materials, such as embalming substances, plugs or plants, were introduced into the body cavities, as seen in other post-medieval mummies [5, 74, 78]. The mummified skin was dry, of leathery appearance and grey or brown colour with no observable embalming substance on it. Overall, the mummies are light due to desiccation. Therefore, it is assumed that the preservation of the bodies from Sommersdorf was due to natural mummification.

Natural mummification can occur under a variety of conditions, such as in very hot, arid and/or cold environments. Besides, it can occur in built environments, including attics and churches [79, 80]. Constant airflow as well as low humidity promote natural mummification due to desiccation that progresses faster than decomposition and thus inhibits bacterial decay [10, 81–83]. The crypt protects the coffins from rain and seasonal variation in temperature, and the parapets are well ventilated through the crenels. Furthermore, ventilation does not only funnel through the crenels but also transversely through the corridors of the parapets (Figs 1 and 2). Therefore, the overall preservation of the mummies is good. Contrarily, the crenels are reaching towards a small lake which brings humidity into the crypt. The effect could be seen in individuals A, B and C who were affected by mycelium of mould fungus prior to conservation, as described by Piñar et al. [84].

Most of the coffins were standing on feet in the form of compressed balls so that direct contact with the ground was avoided and the air could circulate around the coffin. Mummification was presumably enhanced through the coffins’ filling with wood shavings from hardwood. They were produced as waste during the manufacture of the coffin and therefore consist of the same material, mostly oak. Those wood shavings are able to absorb body liquids quite effectively and were typically used as coffin furnishing in the 18^th^ and 19^th^ centuries [69, 72, 85]. Furthermore, oak wood contains tannins that are believed to have a conservative effect on human body tissue and were already used for embalming in Ancient Egypt [86]. Their ability to create solid bonds with proteins ultimately leads to a prevention of further putrefaction. Other burial vaults in Bavaria show the same combination of a skilfully devised ventilation system and absorbent as well as anti-bacterial and anti-mycotic coffin furnishing, namely the crypts of Wald [87], Kalbensteinberg [88] and Sulzbürg [4, 89].

In addition, it was favourable that most of the individuals were entombed within three or four days after death (Table 2), probably before the onset of signs of putrefaction [90]. In this way, they were quickly placed in a climatic environment that led to the desiccation of the corpse. Only two individuals have a deferred interment, namely Agatha Magdalena von Crailsheim (1659–1713) and Wilhelmine Charlotta von Soden (1721–1766) who were entombed several weeks after their death. The reason for this is unclear as they did neither die far from home nor was there a lack of family members to organize the funeral.

Whether or to what extent the above-mentioned preservative conditions were intentionally produced or developed as an unintended (though accepted) consequence of crypt-burial in the post-medieval period is still unclear [91].

### Physical anthropological investigation & mtDNA analysis

If all 13 individuals from Table 2 were actually interred within the crypt, four inhumations would be children, four adult males and five adult females. When comparing the historical records to the physical remains, the original number is difficult to assess and shows discrepancies within and between the sources. The description of Sommersdorf Castle by Josef Bergmann in 1833 mentions only six adult and four children coffins. This number would fit very well to the current number of coffins, considering that coffin lid no. 7 might be a replacement. While the children remain unquestioned throughout all the different sources, the number of adult individuals ranges from six to nine individuals. Physical anthropology detected the remains of a minimum number of nine individuals still preserved in the crypt, among them eight adults, and therefore demands a careful annotation of the registries.

Five out of eight adult individuals are females, three are adult males, and one mummy is an infant. This distribution differs from what is known from other post-medieval crypts which usually contain a high number of child inhumations, reflecting the high infant mortality during the post-medieval period even in aristocratic families [6, 74]. Even though the number of infants is underestimated due to the empty coffins which were found, the family chronicle states that parents lost several children during life [13]. Their absence in the crypt can be explained by the fact that they were probably buried elsewhere, namely in the cemeteries of the family’s residences in Ansbach or Rügland.

The mtDNA analysis of individuals A, C, D, E and G revealed that they are not maternally related. Therefore, the investigated individuals cannot represent both Helena Friderica von Rauber and her daughter Wilhelmine Charlotte von Soden (Table 2) but possibly one or neither of them.

One limitation of the PCR based approach is that there is no distinction between endogenous ancient DNA and possible external modern contamination. Importantly, the results for two individuals could be replicated in a second laboratory which supports authenticity of the retrieved results. In addition, the analysis was undertaken on different samples than the previous investigation but provided the same results. Future aDNA analysis should however include next-generation sequencing (NGS) methodology.

The genetic data can be explained by historical knowledge of pre-modern family structure: First, the daughters of an aristocratic family usually married outside the family and thus lived and died at their husband’s estate. Only when unmarried or widowed, women stayed at their place of birth. Women marrying into a family would have a different mtDNA lineage and would share their mtDNA only with the mutual children that they had with their husbands. Additionally, men often married again after they had lost their first wives during childbirth or due to illnesses. Therefore, it is not surprising that the adult individuals do not share a common female ancestor.

Regarding the predominance of females in comparison to male individuals, it should further be noted that noble men often died far from home in battles and were potentially buried elsewhere. In those cases, however, the widows either returned to the family they were born in, as seen for example for Helena Friderica von Rauber, or—if still young enough—married again. Otherwise, they moved into one of their children’s households.

### Health & socioeconomic status

Body height can be used as a proxy for nutrition and health during adolescence even though the basic range largely depends on genetics. With an average stature of 169.3 cm for males (n = 3) and 163.4 cm for females (n = 5), the Sommersdorf individuals fit very well into the frame given by larger medieval and post-medieval series in Bavaria [92–96] (Table 5). Due to the estimation of body height using the femur, the male individuals from Sommersdorf seem to be smaller than the Bavarian average while the female individuals are taller. However, these differences might be caused by the small sample size and/or decreased deviation through relying on one measurement per individual. Most of the series contain upper- and middle-class status individuals like nobles or monks, except for an urban late medieval poor house cemetery in Regensburg [96].

**Table 5:** Average body height of medieval and post-medieval series from Bavaria (in cm). All estimations were made according to Breitinger 1937 [49] and Bach 1965 [50].

Site	Dating (in centuries)	Males	Females	Reference
Petersberg (monastery, laymen)	10^th^-15^th^	170 (n = 65)	161 (n = 24)	Lösch (2009) [93]
Regenburg (poor house)	12^th^-16^th^	169.6 (n = 143)	160.8 (n = 120)	von Heyking (2013) [96]
Unterregenbach (church)	15^th^-19^th^	172 (n = 2)	161 (n = 17)	Preuschoft and Schneider (1969) [94]
Sulzbürg St. Michael (crypt)	17^th^-18^th^	173 (n = 1)	148 (n = 2)	Lösch (2009) [93]
Sommersdorf (crypt)	17^th^-19^th^	169.3 (n = 3)	163.4 (n = 5)	This study
Attel (monastery)	18^th^	171 (n = 19)	/	Nerlich et al. (2015) [92]

Dental health was examined macroscopically and by computed tomography. Alterations were mainly caused by caries, defined as an irreversible and progressive destruction of the hard dental tissue due to acids produced by micro-organisms of the oral flora [97]. To a lesser degree, dental wear or trauma can make the dentition prone to carious lesions. Other periodontal difficulties, such as abscesses, may follow. The affected tooth will fall out and the alveolus will be remodelled, as it was the case in 27.2% of the teeth in the sample. In less favourable cases, an inflammation develops and spreads to the surrounding teeth and bone. In the current series, in all observable cases the adult individuals had at least one destructive periodontal alteration. Even though the sample size is very small and only lesions of a certain size could be considered due to their representation on the CT images, a caries intensity of 15.6% (or 36% respectively) is within the range for medieval and post-medieval times [96, 98]. However, 100% of the examined individuals with dentition showed at least one carious tooth. The data suggest that the members of the Crailsheim family were consuming a cariogenic diet and not maintaining effective oral hygiene. It remains a task for future research to determine the amount of animal protein in the individuals’ diet [99] which might distinguish the aristocratic family from their tenants who probably mostly relied on carbohydrates. The diet composition could be compared to the mummies’ physique as some show clear signs of overweight.

The curvatures of the spine observed in individual A (Fig 7) and C (Fig 12) show vertebral rotation, no narrowing of the intervertebral space and curvature over several spinal segments [100]; as a result, the pathologies can be diagnosed as scoliosis [101]. There are three types of scoliosis which are all progressive: congenital, idiopathic and neuromuscular. While congenital scoliosis is already present at birth due to a malformation of the vertebrae in utero [102], idiopathic scoliosis develops during childhood and is of unknown aetiology. It is the most common type of scoliosis (80%) and seems to involve genetic and environmental factors [54]. Scoliosis might also develop in advanced age (especially in women above 40 years of age) when vertebral bodies are collapsing due to osteoporosis and attrition of the intervertebral disks. Neuromuscular scoliosis results from weakening of the spinal muscles by neurological disorders such as poliomyelitis, cerebral paralysis or muscular dystrophy. In the present cases, no hints were found for congenital and neuromuscular scoliosis as there are neither structural vertebral defects nor signs of other disorders in the skeletons. As is typical for idiopathic scoliosis, the affected individuals are middle-aged females and show pronounced curvature in the lumbar and thoracic spine, probably causing back pain and reduced mobility. Secondary degenerative changes to the vertebrae in individual A and C indicate an early onset of the disease, possibly during late infancy or adolescence. The specific deviation of the spine from the sagittal plane according to Cobb [103] was not measured as the individuals are lying on their backs and may have undergone post-mortem changes to the position of the spine.

While scoliosis seems to be only marginally affected by social status or diet, it is a common finding in high-status individuals, including mummies [28] and skeletons [74, 104, 105]. Female individuals are more often affected than males.

The high incidence of scoliosis and spinal variations in the present series could be a hint for a genetic relation of the individuals even though anatomical variations of the lumbar vertebrae are very common and primarily without symptom [106]. Symptoms, especially lower back pain, can originate from the anomalous articulation itself in consequence of instability or early degeneration of the joints, or from compressed nerve roots.

In two individuals (A, C), possible haemangiomas were found in the lumbar vertebrae. Those benign vascular tumours are usually asymptomatic. Females are more often affected than males. However, they are still a rare finding in mummy studies [28].

The individuals with supernumerary vertebrae (A, B) or defects of differentiation (D) showed degenerative changes in the spine (Figs 5 and 7). The mtDNA analysis excluded maternal relationship between the individuals A, C and D but could not exclude a paternal line and therefore a paternal predisposition for these variations.

Thoracic calcifications occur in a broad variety of pathologic conditions [107]. Nodule calcifications in the lung are commonly caused by granuloma formation, usually in response to an inflammatory disease, for example tuberculosis [108]. However, other infections, occupational diseases and metastatic calcifications have to be taken into account and demand precise localisation and distribution patterns of the calcifications. On the other hand, calcifications of the aorta and other arteries are commonly interpreted as signs of atherosclerosis [109–111]. The calcifications of individual B are rounded and localized and do not follow arteries’ courses (Fig 9). Due to young adult age and slender constitution, the calcifications might therefore rather represent signs of a chronic inflammation. None of the other individuals showed major calcifications in the blood vessels.

None of the adult individuals who were investigated through CT or by macroscopic examination shows severe degeneration of the larger joints. Besides age, this might be related to the men’s occupation as advisers and administrative officers for the margraves of Brandenburg-Ansbach which did not demand heavy physical activity. Their wives were responsible for the organisation of the household and education of the children.

### Hypotheses on the identification

#### Children

During the late 18^th^ century, the death register lists only four children that were buried in the crypt (Table 2), namely those of Julius Wilhelm von Crailsheim the Elder (1736–1805) and his wife Karolina Eleonora von Reumont (1744–1809). While the parents were later buried in Rügland [13], their children were transported from Ansbach to the family crypt in Sommersdorf. Two boys died some days after birth (Table 2) and were probably interred in the two smallest coffins with 72 respectively 73 cm length (coffins no. 8 and 9) (Table 1). The girls, however, survived longer. It can be assumed that the largest child coffin of 100 cm length (coffin no. 10) was dedicated to the oldest sister Charlotta Eleonora Carolina von Crailsheim (died at 1 year 4 months). The intermediate coffin of 86 cm length (coffin no. 6) was probably meant for the younger sister Karolina Elisabetha Christiana Charlotta von Crailsheim (died at 4 months).

The preserved infant mummy is that of a girl between 3 and 12 months of age and a body height of approx. 67 cm. It is evident that the body must have been relocated in the past since the current coffin no. 9 would have been too small (Fig 15). The overall coffin lengths suggest that the mummy would have fitted best in coffin no. 6 and therefore, the mummy can be identified as 4-months old Karolina Elisabetha Christiana Charlotta von Crailsheim.

All the above-mentioned children died from *Gefraisch* according to the death register (Table 2). *Gefraisch* is an old medical term for convulsions and is believed to be one of the most common causes of death of infants between 3 and 6 months during the early modern period [112]. Even though it is rather a symptom than an actual disease [113], these convulsions were often accompanied by diarrhoea and vomiting which shortly alleviated pain for the children [114]. However, if the convulsions continued due to malnourishment, parasites, fever or injuries, the children often died because of dehydration. In those cases, convulsions were caused by a lack of minerals and vitamins. In other cases, convulsions might have been a symptom of a tetanus infection.

In case of Karolina Elisabetha Christiana Charlotta von Crailsheim, the death register further states the diagnosis *catarrh*. Catarrh is understood as an inflammation of the mucous membranes of the airways or sinuses and is together with sniffing and coughing a symptom of the common cold, pharyngitis or chesty cough (and various other diseases).

#### Male individuals

According to Bergmann, individual B is *Baron von Holz* who fought against the Swedish during the Thirty Years’ War (Fig 3). It is not unlikely that a member of the aristocratic family *von Holz* was buried in Sommersdorf as there were close bonds between both houses. For instance, the godfather of Georg Wolff von Crailsheim was General Georg Friedrich vom Holtz (1597–1666). Even though several branches of the family von Holz [65] were studied, no individual can be considered to be the mummy from Sommersdorf, mostly because of deviating burial places. Likewise, no person of this name was found in the death register of Sommersdorf parish but as this register only starts in 1678, the individual might not have been traced due to death prior to this date. It is also possible that this individual belongs to a completely different family. The individual’s death must have occurred in the second half of the 17^th^ century. The dating of the boots and the anthropological examination (age 25–40 years) exclude an identification as Georg Wolff von Crailsheim (1655–1717), Heinrich Gabriel von Soden (1713–1761) or Julius Wilhelm von Crailsheim (1764–1812).

In the lithograph from 1833, the other completely mummified male individual is presented as a member of the Crailsheim family (Fig 3). Due to the hairstyle and stockings, the portrayed individual can be identified as individual D in coffin no. 4. Sex and age at death of individual D leave Julius Wilhelm von Crailsheim and Heinrich Gabriel von Soden as possible candidates. The coffin dates to the end of the 18^th^ or the beginning of the 19^th^ century. Due to the presence of original wood shavings it seems that coffin and corpse were not commingled in the past, maybe because the inhumation took place after the first looting of the crypt by Napoleonic troops in 1806. In this case, the ascribed identity would match the identification through historical, archaeological and anthropological means. However, the family chronicle mentions a shot in the head during a hunt as possible cause of death for Julius Wilhelm von Crailsheim [13] even though both the death register entry and the chronicle contradict it in the very next sentence (Table 2). No signs of trauma related to an accident as mentioned above were observed for individual D, but also in none of the other male individuals.

The remaining male individual F died at about 40 to 60 years of age. If believing in the previous identification suggested by Bergmann in 1833, individual F either represents Heinrich Gabriel von Soden (1713–1761) or Georg Wolff von Crailsheim (1655–1717). Both are plausible candidates, but no imaging data for this individual are available. Computed tomography or digital X-ray could help to narrow down the age estimation in the future.

As stated above, the possibility of another place of interment than the baronial crypt is also given which leaves it open whether Georg Wolff von Crailsheim was actually buried at the same location as the other men.

#### Female individuals

For the five female individuals identification is even more difficult as they all died at an advanced but similar age. According to the death register, two women died between 40 and 50, two between 50 and 60 and one between 60 and 70 years (Table 2). Due to the varying quality and criteria of the anthropological age estimation, it is challenging to identify the female individuals with certainty. Nevertheless, the individuals C and H seem to represent the youngest women while individual A could be the oldest. It has to be taken into account that computed tomography was only carried out on individual A and C whereas age estimation relied on the state of the dentition for individual E and H. The scattered bones in coffins no. 6 and 10 probably belong to the same female individual but age estimation relied on the degeneration of the joints.

According to Bergmann, two of the portrayed female mummies that correspond to individual A and H (Fig 3) are members of the *von Crailsheim* family. Whether these women were née von Crailsheim, like Helena Friderica von Rauber, or married into the family, like Agatha Magdalena von Hüffel, remains unresolved. Other possible candidates cannot be excluded as we might question the author’s knowledge of the church register and castle’s history.

The third female individual portrayed by Bergmann is individual C (Figs 10 and 11). He claims that this individual was buried alive and that her fingers are therefore still clawed in the chest. The family chronicle identifies her as Sophie Luise von Kniestätt (1649–1690), née von Crailsheim, who died in childbed after giving birth to her eighth child [13]. According to the death register, the stillborn son was buried some days before his mother. The local priest Johann Christian Bernhold (1648–1724) reports in his biography that she suffered from *Mutterfraisch* which might be understood as febrile convulsions. Furthermore, she was partly unconscious and her speech was slurred. His descriptions of symptoms possibly point to eclampsia or other puerperal infections.

Individual C was anthropologically determined as female, aged 30 to 50 years. The pronounced breasts and soft tissue as well as the fat apron on the buttocks suggest an elevated weight and well-nourishment. The positioning of the arms suggests that the hands were originally placed on the breasts but have sunken down with the decomposition and shrinking of the tissue. According to the estimated age, individual C might either be identified as Sophie Luise von Kniestätt (1649–1690) or Wilhelmine Charlotta von Soden (1721–1766). The observed pathologies and particularities are not sufficient to positively identify the mummy.

For this reason, at the current state of research an identification of the female mummies is not possible beyond serious doubts.

## Conclusion

An interdisciplinary approach was used to test the accuracy of historical sources on the mummies from the crypt in Sommersdorf castle. The study revealed that anthropological and mtDNA data match in general with the historical records but there are discrepancies between the numbers of investigated and known entombed individuals. It was, however, surprising that apart from close family members, several likely relatives-in-law were found. The archaeological investigation provided valuable information on the dating of the coffins and the clothes. Computed tomography was essential in narrowing down the age at death range and yielded supplementary data on the health situation of the mummies.

In future studies, the division of historical records into primary and secondary sources will reduce the confusion that family chronicles and oral history may cause.

Through the interplay of different disciplines, probable identification was achieved for Julius Wilhelm von Crailsheim (1764–1812) and his cousin Karolina Elisabetha Christiana Charlotta von Crailsheim (1769–1770). For the other individuals, identification was hampered by the fact that the crypt was looted and re-arranged in the past.

The study highlights the strengths and weaknesses of different methodological approaches in bioarchaeological research and calls for awareness of the limitations of each record. It also demonstrates the benefit of synoptical evaluation and mutual validation of various approaches.

## Supporting information

S1 File List of used archival records. (DOCX)

S2 File mtDNA analysis protocol. (DOCX)

## References

[1] Tingle EC, Willis J. Dying, Death, Burial and Commemoration in Reformation Europe. London; New York: Routledge; 2016.

[2] Käpplinger J. Die Särge der fränkischen Hohenzollern zu Ansbach und Bayreuth 1603–1791. Regensburg: Schnell & Steiner; 2015.

[3] Schwarz R, Renhart S, Gruber H, Klimesch W, Neuhuber F, Cemper-Kiesslich J. In naming the dead: Autosomal and Y-chromosomal STR typing on human skeletal remains from an 18th/19th century aristocratic crypt in Gallspach, Upper Austria. Anthropol Anz. 2015;72(3):335–346. doi: 10.1127/anthranz/2015/0515 25806829 10.1127/anthranz/2015/0515

[4] Lösch S, Bunzel M, Lehn C, Struck U, Peschel O, Graw M, et al. Die Wolfstein Mumien (16.-18. Jahrhundert)—Untersuchungen an mumifizierten Körpern einer Gruft in Süddeutschland. In: Arbeitsgemeinschaft Friedhof und Denkmal, editor. Geschichte und Tradition der Mumifizierung in Europa. Beiträge zu einer Tagung im Museum für Sepulkralkultur 2010. Kasseler Studien zur Sepulkralkultur. Kassel 2011. pp. 113–119.

[5] Panzer S, Zink AR, Piombino-Mascali D. Scenes from the Past: Radiologic Evidence of Anthropogenic Mummification in the Capuchin Catacombs of Palermo, Sicily. RadioGraphics. 2010;30(4):1123–1132. doi: 10.1148/rg.304095174 20631372 10.1148/rg.304095174

[6] Binder M, Pany D. Anthropological Analysis of 26 natural mummies (17th/18th century AD) from Schloss Albrechtsberg a. d. Pielach, Lower Austria In: Cemper-Kiesslich J, Lang F, Schaller K, Uhlir C, Unterwurzacher M, editors. archaeoPLUS. Schriften zur Archäologie und Archäometrie der Paris Lodron-Universität Salzburg 1 Salzburg 2010 pp. 42–48.

[7] Pap I, Susa E, Józsa L. Mummies from the 18th–19th century Dominican Church of Vác, Hungary. Acta Biol Szeged. 1997;42:107–112.

[8] Cox M. Crypt Archaeology: an Approach. Reading 2001.

[9] Fingerlin I. Die Grafen von Sulz und ihr Begräbnis in Tiengen am Hochrhein Forschungen und Berichte der Archäologie in Baden-Württemberg 15 Stuttgart: Konrad Theiss Verlag; 1992.

[10] Petrella E, Piciucchi S, Feletti F, Barone D, Piraccini A, Minghetti C, et al CT Scan of Thirteen Natural Mummies Dating Back to the XVI-XVIII Centuries: An Emerging Tool to Investigate Living Conditions and Diseases in History. PLOS ONE. 2016;11(6):e0154349 doi: 10.1371/journal.pone.0154349 27355351 10.1371/journal.pone.0154349 PMC4927149

[11] Ramisch HK. Landkreis Feuchtwangen. Kurzinventar Bayerische Kunstdenkmale 21 München: Deutscher Kunstverlag; 1964.

[12] Gräser H. Die Freiherren von Crailsheim—ein fränkisches Rittergeschlecht. Triesdorfer Hefte. 1990;3:1–18.

[13] von Crailsheim SF. Die Reichsfreiherrn von Crailsheim: Familiengeschichte Nach dem Stande von 1904. München: Straub; 1905.

[14] von Crailsheim M Baron. The Mummies from Sommersdorf Castle In: Wieczorek A, Rosendahl W, editors. Mummies of the World. Munich, Berlin, London, New York: Prestel; 2010 pp. 362–364.

[15] Gill-Frerking H. Crypt mummies of Sommersdorf Castle In: Cardin M, editor. Mummies around the World: An Encyclopedia of Mummies in History, Religion, and Popular Culture. Santa Barbara; Denver; Oxford: ABC-CLIO; 2015 pp. 81–83.

[16] Wieczorek A, Tellenbach M, Rosendahl W. Mumien Der Traum vom ewigen Leben. Mainz: Philipp von Zabern; 2007.

[17] Wieczorek A, Rosendahl W. Mummies of the World. Munich, Berlin, London, New York: Prestel; 2010.

[18] Gill-Frerking H, Schanandore JV, Rosendahl W. Supernumerary vertebrae and other spinal pathology in three 17th century crypt mummies from Germany. Poster, 7th World Congress on Mummy Studies; San Diego 2011.

[19] Rechter G, Wyschkon J. Die Archive der Familienstiftung von Crailsheim. Familienkonsulentie und Herrschaft Rügland Altes und Neues Archiv I & II. München: Generaldirektion der Staatlichen Archive Bayerns; 2007.

[20] Biedermann JG. Geschlechts-Register der Reichs-Frey unmittelbaren Ritterschafft Landes zu Francken, löblichen Orts Steigerwald, welches aus denen bewährtesten Urkunden, Kauf-Lehen- und Heyraths-Briefen, gesammelten Grabschriften und eingeholten genauen Nachrichten von innen beschriebenen Gräflich-Freyherrlich- und Edlen Häusern in Gegenwärtige Ordnung verfasset und richtig zusammen getragen worden. Nürnberg: Franz Köngott; 1748.

[21] Tyroff K. Geschlechts- und Wappenbeschreibungen zu dem Tyroffischen neuen adelichen Wappenwerk. Nürnberg: Conrad Tyroffisches Wappencomtoir; 1805.

[22] Grieb MH. Nürnberger Künstlerlexikon. Bildende Künstler, Kunsthandwerker, Gelehrte, Sammler, Kulturschaffende und Mäzene vom 12. bis zur Mitte des 20 Jahrhunderts. München: K. G. Saur; 2007.

[23] Ströbl A. Die Entwicklung des Holzsarges von der Hochrenaissance bis zum Historismus im nördlichen und mittleren Deutschland Kasseler Studien zur Sepulkralkultur 20 Kassel: Fachverlag des deutschen Bestattungsgewerbes 2014.

[24] Brückner W. Ein Frauengrab von 1758 in der Kirche zu Nennslingen, Landkreis Weissenburg-Gunzenhaus, Mittelfranken. Das Archäologische Jahr in Bayern. 1984;1983:183–187.

[25] Lenk N. Die Restaurierung der Holzsärge In: Oehmig S, editor. Die Restaurierung der Wolfstein-Gruft in der ev. Schlosskirche St Michael, Sulzbürg: Beiträge zur Restaurierung 2002–2008. Wasserburg: Institut für Restaurierung; 2008 pp. 19–22.

[26] Klocke J. Conservation of Mummies—Having Bones to Pick with the Dead In: Wieczorek A, Rosendahl W, editors. Mummies of the World. Munich, Berlin, London, New York: Prestel Verlag; 2010 pp. 251–253.

[27] Panzer S, Mc Coy MR, Hitzl W, Piombino-Mascali D, Jankauskas R, Zink AR, et al Checklist and Scoring System for the Assessment of Soft Tissue Preservation in CT Examinations of Human Mummies. PLOS ONE. 2015;10(8):e0133364 doi: 10.1371/journal.pone.0133364 26244862 10.1371/journal.pone.0133364 PMC4526695

[28] Piombino-Mascali D, Kozakaitė J, Tamošiūnas A, Valančius R, Panzer S, Jankauskas R. Skeletal pathological conditions of Lithuanian mummies. Papers on Anthropology. 2014;23(1):118–126. doi: 10.12697/poa.2014.23.1.10

[29] Aufderheide AC. The Scientific Study of Mummies. Cambridge: Cambridge University Press; 2003.

[30] Rühli FJ, Chhem RK, Böni T. Diagnostic paleoradiology of mummified tissue: interpretation and pitfalls. Can Assoc Radiol J. 2004;55(4):218–227. 15362344

[31] Acsádi G, Nemeskéri J. History of Human Lifespan and Mortality. Budapest, Hungary: Akadémiai Kiadó; 1970.

[32] Buikstra JE, Ubelaker DH. Standards for data collection from human skeletal remains: Proceedings of a seminar at the Field Museum of Natural History, organised by Jonathan Haas. Fayetteville, Arkansas: Arkansas Archeological Survey; 1994.

[33] Grabherr S, Cooper C, Ulrich-Bochsler S, Uldin T, Ross S, Oesterhelweg L, et al Estimation of sex and age of “virtual skeletons”–a feasibility study. Eur Radiol. 2009;19(2):419–429. doi: 10.1007/s00330-008-1155-y 18766348 10.1007/s00330-008-1155-y

[34] Schanandore JV. Examination of age at death methods and the effects on estimation accuracy when applied to computed tomography scans and virtual models of mummies [Dissertation]. Fargo: North Dakota State University; 2015.

[35] Villa C, Buckberry J, Lynnerup N. Evaluating osteological ageing from digital data. J Anat. 2016 doi: 10.1111/joa.12544 27620700 10.1111/joa.12544 PMC6637452

[36] Brothwell DR. Digging up Bones The excavation, treatment and study of human skeletal remains. 3 ed Oxford: Oxford University Press; 1981.

[37] Miles AEW. Dentition in the assessment of individual age in skeletal material In: Brothwell DR, editor. Dental Anthropology. Symposia of the Society for the Study of Human Biology 5 Oxford, London, UK: Pergamon Press; 1963 pp. 191–209.

[38] Boyd KL, Villa C, Lynnerup N. The Use of CT Scans in Estimating Age at Death by Examining the Extent of Ectocranial Suture Closure. J Forensic Sci. 2015;60(2):363–369. doi: 10.1111/1556-4029.12683 25619969 10.1111/1556-4029.12683

[39] Schultz M. Paläopathologische Diagnostik In: Knussmann R, editor. Anthropologie. Handbuch der vergleichenden Biologie des Menschen, I Wesen und Methoden der Anthropologie. Stuttgart, New York: Gustav Fischer Verlag; 1988 pp. 480–496.

[40] Garvin HM. Ossification of Laryngeal Structures as Indicators of Age. J Forensic Sci. 2008;53(5):1023–1027. doi: 10.1111/j.1556-4029.2008.00793.x 18624888 10.1111/j.1556-4029.2008.00793.x

[41] Leopold D, Schäfer W. Röntgenologische Methoden zur Identifikation In: Leopold D, editor. Identifikation unbekannter Toter. Interdisziplinäre Methodik, forensische Osteologie. Lübeck: Verlag Schmidt-Römhild; 1998 pp. 289–326.

[42] Brooks S, Suchey JM. Skeletal age determination based on the os pubis: A comparison of the Acsádi-Nemeskéri and Suchey-Brooks methods. Hum Evol. 1990;5(3):227–238. doi: 10.1007/bf02437238

[43] Villa C, Buckberry J, Cattaneo C, Lynnerup N. Technical Note: Reliability of Suchey-Brooks and Buckberry-Chamberlain methods on 3D visualizations from CT and laser scans. Am J Phys Anthropol. 2013;151(1):158–163. doi: 10.1002/ajpa.22254 23595646 10.1002/ajpa.22254

[44] Scheuer L, Black S. Developmental Juvenile Osteology. London: Academic Press; 2000.

[45] Stloukal M, Hanáková H. Die Länge der Längsknochen altslawischer Bevölkerungen—unter besonderer Berücksichtigung der Wachstumsfragen. Homo. 1978; 29:53–68.

[46] Schmid F, Künle F. Das Längenwachstum der langen Röhrenknochen in bezug auf Körperlänge und Lebensalter. Fortschr Geb Rontgenstr Nuklearmed. 1958;89:350–356. doi: 10.1055/s-0029-1226163 13586452

[47] Elgenmark O. The normal development of the ossific centres during infancy and childhood. Stockholm 1946.

[48] Martin R. Lehrbuch der Anthropologie in systematischer Darstellung mit besonderer Berücksichtigung der anthropologischen Methoden. Jena: Gustav Fischer 1914.

[49] Breitinger E. Zur Berechnung der Körperhöhe aus den langen Gliedmaßenknochen. Anthropol Anz. 1937;14:249–274.

[50] Bach H. Zur Berechnung der Körperhöhe aus den langen Gliedmaßenknochen weiblicher Skelette. Anthropol Anz. 1965;29:12–21.

[51] Zech W-D, Näf M, Siegmund F, Jackowski C, Lösch S. Body height estimation from post-mortem CT femoral F1 measurements in a contemporary Swiss population. Leg Med. 2016;19:61–66. doi: 10.1016/j.legalmed.2016.02.004 26980256 10.1016/j.legalmed.2016.02.004

[52] Hillson S. Dental Anthropology. Cambridge: Cambridge University Press; 1996.

[53] Roberts C, Manchester K. The Archaeology of Disease. 2 ed Ithaca, New York: lan Sutton Publishing Ltd, Cornell University Press; 1995.

[54] Aufderheide AC, Rodríguez-Martín C. The Cambridge Encyclopedia of Human Paleopathology. Cambridge: Cambridge University Press; 1998.

[55] Ortner DJ. Identification of pathological conditions in human skeletal remains. London: Routledge; 2003.

[56] Anderson S, Bankier AT, Barrell BG, de Bruijn MHL, Coulson AR, Drouin J, et al Sequence and organization of the human mitochondrial genome. Nature. 1981;290:457 doi: 10.1038/290457a0 7219534 10.1038/290457a0

[57] Andrews RM, Kubacka I, Chinnery PF, Lightowlers RN, Turnbull DM, Howell N. Reanalysis and revision of the Cambridge reference sequence for human mitochondrial DNA. Nat Genet. 1999;23(2):147–147. doi: 10.1038/13779 10508508 10.1038/13779

[58] Lee HY, Song I, Ha E, Cho S-B, Yang WI, Shin K-J. mtDNAmanager: a Web-based tool for the management and quality analysis of mitochondrial DNA control-region sequences. BMC Bioinformatics. 2008;9:483 doi: 10.1186/1471-2105-9-483 19014619 10.1186/1471-2105-9-483 PMC2621369

[59] Alterauge A. Crypt Mummies from the 17th to the 19th Century in Middle Franconia—An Interdisciplinary Investigation. The Society for Historical Archaeology Newsletter. 2015;48(1):28–30.

[60] Adler C-P. Bone Diseases: Macroscopic, Histological, and Radiological Diagnosis of Structural Changes in the Skeleton. Heidelberg; Berlin: Springer; 2000.

[61] Mann RW, Hunt DR. Photographic Regional Atlas of Bone Disease: A Guide to Pathologic and Normal Variation in the Human Skeleton. Springfield, Illinois: Charles C Thomas; 2005.

[62] Stloukal M. Der Gesundheitszustand des Gebisses bei der Population von Grossmährischen Mikulčice. Anthropologie. 1963;1:35–45.

[63] Brotherton P, Haak W, Templeton J, Brandt G, Soubrier J, Jane Adler C, et al Neolithic mitochondrial haplogroup H genomes and the genetic origins of Europeans. Nat Commun. 2013;4:1764 doi: 10.1038/ncomms2656 23612305 10.1038/ncomms2656 PMC3978205

[64] von Ow MF. Christoph Wilhelm Freiherr von Rauber, 23 Jahre als Häftling auf der Wülzburg. Jahrbuch des Historischen Vereins für Mittelfranken. 1985;1984(92):289–298.

[65] vom Holtz MGF. Generalfeldzeugmeister Georg Friedrich vom Holtz auf Alfdorf, Hohenmühringen, Aichelberg u.s.w. Ein Lebensbild aus dem 17. Jahrhundert. Stuttgart 1891.

[66] Müller A. Kirchenbücher als wissenschaftliche Quelle. Zeitschrift für Bayerische Kirchengeschichte. 2002;71:223–235.

[67] Alterauge A, Bodenstein J, Streitz C, Friske M, Rosendahl W. Zwei Erbbegräbnisse des 18. Jahrhunderts in der Westhalle der St. Katharinenkirche zu Salzwedel—Archäologische und anthropologische Untersuchungsergebnisse zum Totenbrauchtum in der Altmark In: Simitopoulou K, Zafeiris K, Theodorou T, Papageorgopoulou C, editors. Anthropological Pathways. Festschrift for Professor NI Xirotiris. Komotini: Mystis; 2014 pp. 119–131.

[68] Kenzler H. Religion, Status and Taboo. Changing Funeral Rites in Catholic and Protestant Germany In: Tarlow S, editor. The Archaeology of Death in Post-medieval Europe. Warsaw; Berlin: De Gruyter; 2015 pp. 148–169.

[69] Steeger W. Zum Wandel der Begräbnisform vom Frühmittelalter bis zum 17. Jahrhundert am Beispiel archäologischer Funde in Bad Windsheim. In: Thurnwald AK, editor. Trauer und Hoffnung. Sterbebräuche, Totengedenken und Auferstehungsglauben in evangelischen Gemeinden. Schriften und Kataloge des Fränkischen Freilandmuseums. Bad Windsheim: Verlag Fränkisches Freilandmuseum; 2003. pp. 11–39.

[70] Swann J. Shoes. London: Batsford; 1983.

[71] Goubitz O. Stepping Through Time: Archaeological Footwear from Prehistoric Times Until 1800. Zwolle: SPA Uitgevers; 2007.

[72] Fehring GP, Stachel G. Archäologische Untersuchungen in der Stadtkirche St. Johannes d.T. zu Crailsheim In: König H-J, editor. Die Johanneskirche in Crailsheim. Kirchberg/Jagst: Wettin; 1967 pp. 9–36.

[73] Jackowski C, Bolliger S, Thali MJ. Common and unexpected findings in mummies from ancient Egypt and South America as revealed by CT. RadioGraphics. 2008;28(5):1477–1492. doi: 10.1148/rg.285075112 18794321 10.1148/rg.285075112

[74] Mylius U. Pathologisch-anthropologische Untersuchungen einer Adelsfamilie des 17. Jahrhunderts: Ausgrabung 1978 in der Fürstengruft der Pfarrkirche "Maria Himmelfahrt" in Tiengen am Hochrhein [Dissertation]. Freiburg i. Br.: Universität Freiburg; 1984.

[75] Colleter R, Dedouit F, Duchesne S, Mokrane F-Z, Gendrot V, Gérard P, et al Procedures and Frequencies of Embalming and Heart Extractions in Modern Period in Brittany. Contribution to the Evolution of Ritual Funerary in Europe. PLOS ONE. 2016;11(12):e0167988 doi: 10.1371/journal.pone.0167988 28030554 10.1371/journal.pone.0167988 PMC5193353

[76] Piombino-Mascali D, Jankauskas R, Zink AR, Sergio Todesco M, Aufderheide AC, Panzer S. Paleoradiology of the Savoca Mummies, Sicily, Italy (18th–19th Centuries AD). Anat Rec. 2015;298(6):988–1000. doi: 10.1002/ar.23132 25998633 10.1002/ar.23132

[77] Pinheiro J. Decay Process of a Cadaver In: Schmitt A, Cunha E, Pinheiro J, editors. Forensic Anthropology and Medicine. Complementary Sciences from Recovery to Cause of Death. Totowa: Humana Press; 2006 pp. 85–116.

[78] Kristóf LA, Pohárnok L, Kerényi T, Istók R, Hargittai P, Tóth G, et al. Paleoradiological and paleopatholgical examinations of a 300 years old Hungarian archbishop’s mummy. Poster, The European Congress of Radiology (ECR); Vienna 2010.

[79] Schoenen D. Verwesung Der mikrobielle Abbauprozess menschlicher Leichen und seine Bedeutung für den Öffentlichen Gesundheitsdienst, Hygiene, Friedhofswesen, Bodenkunde, Rechtsmedizin und Kriminologie. Aachen: Shaker Verlag; 2013.

[80] Szikossy I, Kustár A, Guba Z, Kristóf LA, Pap I. Naturally Mummified Corpses from the Dominican Church in Vác, Hungary In: Wieczorek A, Rosendahl W, editors. Mummies of the World. Munich, Berlin, London, New York: Prestel; 2010 pp. 160–171.

[81] Kleiss E. Zum Problem der natürlichen Mumifikation und Konservierung. Z Morphol Anthropol. 1967;59(2):204–213. 5605131

[82] Aturaliya S, Lukasewycz A. Experimental forensic and bioanthropological aspects of soft tissue taphonomy: 1. Factors influencing postmortem tissue desiccation rate. J Forensic Sci. 1999;44(5):893–896. 10486936

[83] Saukko P, Knight B. Knight's Forensic Pathology. 3 ed London 2004.

[84] Piñar G, Piombino-Mascali D, Maixner F, Zink A, Sterflinger K. Microbial survey of the mummies from the Capuchin Catacombs of Palermo, Italy: biodeterioration risk and contamination of the indoor air. FEMS Microbiol Ecol. 2013;86(2):341–356. doi: 10.1111/1574-6941.12165 23772650 10.1111/1574-6941.12165 PMC3916889

[85] Ströbl A, Vick D. Hopfenbett und Hexenkraut. Oder: Wie christlich ist Aberglaube? In: Beilke-Voigt I, Biermann F, editors. Glaube—Aberglaube—Tod. Vom Umgang mit dem Tod von der Frühgeschichte bis zur Neuzeit. Ethnographisch-Archäologische Zeitschrift. Berlin 2009 pp. 311–326.

[86] Tchapla A, Méjanelle P, Bleton J, Goursaud S. Characterisation of embalming materials of a mummy of the Ptolemaic era. Comparison with balms from mummies of different eras. J Sep Sci. 2004;27(3):217–234. doi: 10.1002/jssc.200301607 15334909 10.1002/jssc.200301607

[87] Behrend J-P, Schmitz E. Mumien. Botschafter fürs Jenseits. 5000 Jahre Totenkult In: Kirchner G, editor. Terra-X: Expeditionen ins Unbekannte Mumien, Magier, Meuterer. München: Keyser 1993 pp. 230–271.

[88] Spoerl W. Rieter-Kirche Kalbensteinberg. Kalbensteinberg 1980.

[89] Graw M, Holley S, Zink A, Peschel O. Forensisch-anthropologische Untersuchungen In: Oehmig S, editor. Die Restaurierung der Wolfstein-Gruft in der ev. Schlosskirche St Michael, Sulzbürg: Beiträge zur Restaurierung 2002–2008. Wasserburg 2008 pp. 27–32.

[90] Krause D. Späte Leichenveränderungen In: Brinkmann B, Madea B, editors. Handbuch gerichtliche Medizin. 1 Berlin, Heidelberg: Springer; 2004 pp. 150–226.

[91] Schmitz-Esser R. Der Leichnam im Mittelalter Einbalsamierung, Verbennung und die kulturelle Konstruktion des toten Körpers. Ostfildern: Jan Thorbecke Verlag; 2014.

[92] Nerlich AG, Riepertinger A, Gillich R, Panzer S. Paleopathology and Nutritional Analysis of a South German Monastery Population. Biomed Res Int. 2015;2015:ID 486467. doi: 10.1155/2015/486467 26347256 10.1155/2015/486467 PMC4545279

[93] Lösch S. Paläopathologisch-anthropologische und molekulare Untersuchungen an mittelalterlichen und frühneuzeitlichen Bevölkerungsgruppen. Ernährung und Gesundheitszustand süd- und nordbayerischer Bevölkerungsstichproben [Dissertation]. München: Ludwig-Maximilians-Universität 2009.

[94] Preuschoft H, Schneider H. Die Skelettreste aus der Ev. Pfarrkirche St. Veit zu Unterregenbach (Gde. Langenburg, Württemberg). Anthropologie. 1969;7(1):55–69.

[95] Haidle MN. A multiple myeloma of the late middle ages from Unterregenbach, southwestern Germany. Int J Osteoarchaeol. 1995;5(4):359–367.

[96] von Heyking K. Anthropologie einer frühstädtischen Randgruppe: morphologische und archäometrische Untersuchung eines hoch- bis spätmittelalterlichen Armenhausgräberfeldes in Regensburg [Dissertation]. München: Ludwig-Maximilians-Universität 2013.

[97] Caselitz P. Caries—Ancient Plague of Humankind In: Alt KW, Rösing FW, Teschler-Nicola M, editors. Dental Anthropology. Fundamentals, Limits, and Prospects. Wien: Springer; 1998 pp. 203–226.

[98] Mant M, Roberts C. Diet and Dental Caries in Post-Medieval London. Int J Hist Archaeol. 2015;19(1):188–207. doi: 10.1007/s10761-014-0286-x

[99] Haeusler M, Haas C, Lösch S, Moghaddam N, Villa IM, Walsh S, et al Multidisciplinary Identification of the Controversial Freedom Fighter Jörg Jenatsch, Assassinated 1639 in Chur, Switzerland. PLOS ONE. 2016;11(12):e0168014 doi: 10.1371/journal.pone.0168014 28030571 10.1371/journal.pone.0168014 PMC5193413

[100] Mau H. Die Differentialdiagnose der beginnenden Skoliose beim M. Scheuermann gegenüber der idiopathischen Skoliose. Z Orthop Unfall. 1982;120(01):58–63. doi: 10.1055/s-2008-1051576 10.1055/s-2008-1051576 6805150

[101] Janicki JA, Alman B. Scoliosis: Review of diagnosis and treatment. Paediatr Child Health. 2007;12(9):771–776. 19030463 10.1093/pch/12.9.771 PMC2532872

[102] Kilgore L, Van Gerven D. Congenital scoliosis: possible causes and consequences in a skeleton from Nubia. Int J Osteoarchaeol. 2010;20(6):630–644. doi: 10.1002/oa.1085

[103] Cobb JR. Outline for the study of scoliosis. The American Academy of Orthopedic Surgeons Instructional Course Lectures. 1948;5:261–275.

[104] Appleby J, Mitchell PD, Robinson C, Brough A, Rutty G, Harris RA, et al The scoliosis of Richard III, last Plantagenet King of England: diagnosis and clinical significance. Lancet. 2014;383(9932):1944 doi: 10.1016/S0140-6736(14)60762-5 24881996 10.1016/S0140-6736(14)60762-5

[105] Villari N, Fornaciari G, Lippi D, Cerinic MM, Ginestroni A, Pellicanò G, et al The Medici Project: Radiographic Survey. RadioGraphics. 2009;29(7):2101–2114. doi: 10.1148/rg.297085212 19926765 10.1148/rg.297085212

[106] Konin GP, Walz DM. Lumbosacral Transitional Vertebrae: Classification, Imaging Findings, and Clinical Relevance. AJNR Am J Neuroradiol. 2010;31(10):1778–1786. doi: 10.3174/ajnr.A2036 20203111 10.3174/ajnr.A2036 PMC7964015

[107] Brown K, Mund DF, Aberle DR, Batra P, Young DA. Intrathoracic calcifications: radiographic features and differential diagnoses. RadioGraphics. 1994;14(6):1247–1261. doi: 10.1148/radiographics.14.6.7855339 7855339 10.1148/radiographics.14.6.7855339

[108] Piombino-Mascali D, Jankauskas R, Tamosiunas A, Valancius R, Gill-Frerking H, Spigelman M, et al Evidence of probable tuberculosis in Lithuanian mummies. Homo. 2015;66(5):420–431. doi: 10.1016/j.jchb.2015.01.004 26048368 10.1016/j.jchb.2015.01.004

[109] Allam AH, Thompson RC, Wann LS, Miyamoto MI, Nur el-Din Ae-H, el-Maksoud GA, et al Atherosclerosis in Ancient Egyptian Mummies. JACC Cardiovasc Imaging. 2011;4(4):315–327. doi: 10.1016/j.jcmg.2011.02.002 21466986 10.1016/j.jcmg.2011.02.002

[110] Thompson RC, Allam AH, Zink A, Wann LS, Lombardi GP, Cox SL, et al Computed Tomographic Evidence of Atherosclerosis in the Mummified Remains of Humans From Around the World. Glob Heart. 2014;9(2):187–196. doi: 10.1016/j.gheart.2014.03.2455 25667088 10.1016/j.gheart.2014.03.2455

[111] Piombino-Mascali D, Jankauskas R, Tamošiūnas A, Valančius R, Thompson RC, Panzer S. Atherosclerosis in mummified human remains from Vilnius, Lithuania (18th–19th centuries AD): A computed tomographic investigation. Am J Hum Biol. 2014;26(5):676–681. doi: 10.1002/ajhb.22578 24948424 10.1002/ajhb.22578

[112] Steinheimer M. Gesundheitserziehung und -vorsorge zur Zeit der Aufklärung mit einer Untersuchung der Sterbefälle in den Jahren 1780–1785 in der Pfarrei Roßtal. Rosstaler Heimatblätter; Mitteilungen des Heimatvereins Rosstal 1985;11(1):15–25.

[113] Bengtsson M. The Interpretation of Cause of Death Among Infants. Hygiea Internationalis. 2002;3(1):53–73.

[114] Schnitzer A, Wolff B. Handbuch der Kinderkrankheiten. Leipzig: F. A. Brockhaus; 1843.

