# Supplementary material for: The Sommersdorf mummies—An interdisciplinary investigation on human remains from a 17th-19th century aristocratic crypt in southern Germany
Source: PLoS One. 2017 Aug 31;12(8):e0183588. doi: 10.1371/journal.pone.0183588 (PMC5578507; doi:10.1371/journal.pone.0183588)
Supplement: S1 File — (DOCX) [file pone.0183588.s001.docx]

**Supporting Information S1**

**List of used archival records**

Staatsarchiv Nürnberg: Archive der Familienstiftung v. Crailsheim, Herrschaft Sommersdorf-Thann, A94, A184, N3105

Landeskirchliches Archiv der Evangelisch-lutherischen Kirche in Bayern: PfA Sommersdorf, KB 9.5.0001-507-3 & KB 9.5.0001-507-4

Josef Bergmann, Lithographie und Beschreibung der Alten Burg Sommersdorf, 1833 (in possession of Manfred Freiherr von Crailsheim)
