# Supplementary material for: The Sommersdorf mummies—An interdisciplinary investigation on human remains from a 17th-19th century aristocratic crypt in southern Germany
Source: PLoS One. 2017 Aug 31;12(8):e0183588. doi: 10.1371/journal.pone.0183588 (PMC5578507; doi:10.1371/journal.pone.0183588)
Supplement: S2 File — (DOCX) [file pone.0183588.s002.docx]

**Supporting Information S2**

**mtDNA analysis protocol**

**Centre for Evolutionary Medicine, Institute of Anatomy, University of Zurich**

Five DNA samples of individuals A, C, D, E and G were analysed at the Centre for Evolutionary Medicine, Institute of Anatomy at the University of Zurich (today: Institute of Evolutionary Medicine). The anticontamination procedures have been described elsewhere {Krüttli, 2014 #2217}[1]. All bone and tooth samples were cleaned of soft tissue remnants, tooth roots and bone were powdered in a SPEX freezer mill 6770 (SPEX industries, Metuchen, USA) before 400mg of powder was washed in 1% bleach solution and the bleach removed by repeated washes with MilliQ (millipure) water and dried. The cleaned powder was incubated in 1ml extraction solution (0.45M EDTA, 10% Proteinase K) for 12 hours at 55^o^C and 24 hours at room temperature. The resultant solution was passed through Amicon Ultra -0.5ml 30K filters following manufacturer’s instructions and the DNA solution was then concentrated and cleaned using a QIAGEN PCR purification kit (QIAGEN, Venlo, Netherlands). The extractions were performed in two separate batches with at least one extraction blank alongside.

The extracted samples and negative extraction and PCR controls were amplified with 0.2μl Phusion Hot Start II polymerase (Thermo Fisher Scientific, Waltham, USA), 4μl 5X Phusion HF buffer, 2μl of 2mM dNTPs, 1μl of 2.5mg/mL BSA, 1μl of 10 μM forward primer, 1μl of 10 μM reverse primer, and 9.8μl H_2_O, plus 1μl of sample extract for previously published primer sets that cover the Hyper Variable Region I and II (HVR-I, HVR-II) of the control region of the mtDNA genome [2]. Amplicons were cloned into pBluescript KS vectors (Stratagene, La Jolla, USA) following manufacturer’s instructions and then transformed into competent *Escherichia coli* cells before being purified by Mini-prep kit (QIAGEN, Venlo, Netherlands) and sequenced.

The sequences were aligned against the revised Cambridge Reference Sequence (rCRS) using CLC Main Workbench 5 software [3], and the consensus sequence was determined. The HVR-I and II sequences for each sample were distinct from the investigator’s sequence (investigator: NS).

**Institute for Mummy Studies, Eurac research Bolzano**

Two individuals (C and G) were molecularly analysed in the aDNA laboratory in Bolzano, Italy. Sample preparation and DNA extraction was performed in a dedicated pre-PCR area following the strict procedures required for studies of aDNA: use of protective clothing, UV-light exposure of the equipment and bleach sterilization of surfaces, use of PCR workstations and filtered pipette tips. Within a designated sample preparation room the outer surface of the bone samples was mechanically removed by using a Dremel speed rotary tool. The surfaces of the hard tissue material were subsequently subjected to a 15 min UV treatment. Finally, the cleaned samples were pulverized using a Retsch mixer mill (25 Hz, 30 sec). DNA extraction was performed with approximately 250 mg of tissue powder using a silica-based DNA extraction technique described by Rohland et al. [4] with minor modifications. Different primer combinations were used for the analysis of the mitochondrial haplotype (Table S1).

**Table S1: List of the mitochondrial DNA Primers used in this study and the corresponding PCR conditions**

| **Molecular target** | **Primer** | **Sequence (5´-3´)** | **Product (bp)** | **PCR conditions** | **Reference** |
| --- | --- | --- | --- | --- | --- |
| mtDNA HVR-I | L16117 | TACATTACTGCCAGCCACCAT | 162 | 95°C, 5 min; 95°C, 55°C, and 72°C, 45 sec for 38 cycles | [5] |
|  | H16233 | GCTTTGGAGTTGCAGTTGATGTGT |  |  |  |
| mtDNA HVR-I | L16209 | CCCCATGCTTACAAGCAAGT | 133 | 95°C, 5 min; 95°C, 55°C, and 72°C, 45 sec for 38 cycles | [6] |
|  | H16303 | TGGCTTTATGTACTATGTAC |  |  |  |
| mtDNA HVR-I | L16287 | CACTAGGATACCAACAAACC | 162 | 95°C, 5 min; 95°C, 55°C, and 72°C, 45 sec for 38 cycles | [6] |

The PCR reaction mix for all primer sets contained 10 mM tris-HCl (pH 8.3), 50 mM KCl, 1.875 mM MgCl2, 200 µM of each deoxynucleotide triphosphate, 0.5 µM of each primer, 0.1 mg/ml Bovine serum albumin, 0.05 U/µl AmpliTaq Gold (Applied Biosystems, Foster City, CA, USA) and 4 µl of extracted DNA to a final volume of 50 µl. Polymerase chain reaction was carried out according to the parameters in Table S1. The PCR products were separated by electrophoresis on a 2.85 % agarose gel and visualized on a UV-screen after staining with ethidium bromide.

The nucleotide sequences of the PCR products were determined by direct sequencing. The PCR amplification products were purified (EXO-SAP treatment) and four microliters of the PCR amplification cleaned-product were sequenced on an ABI Prism 310 DNA automated sequencer, using the BigDyeTM Terminator Cycle Sequencing Ready Reaction Kit version 3.1 (Applied Biosystems, Foster City, CA, USA). Primers used for the sequencing were the same as those used for the PCRs. Results were compared to the Cambridge reference sequence for human mtDNA (GenBank, accession 251831106. ref.NC_012920.1).

For both individuals (C and G) the same mitochondrial HVR-I motifs were obtained as in the laboratory in Zurich. The results for this part of the mtDNA have been successfully replicated.

**References**

1. Krüttli A, Bouwman A, Akgül G, Della Casa P, Rühli F, Warinner C. Ancient DNA Analysis Reveals High Frequency of European Lactase Persistence Allele (T-13910) in Medieval Central Europe. PLOS ONE. 2014;9(1):e86251. doi: 10.1371/journal.pone.0086251

2. Kemp BM, Malhi RS, McDonough J, Bolnick DA, Eshleman JA, Rickards O, et al. Genetic analysis of early holocene skeletal remains from Alaska and its implications for the settlement of the Americas. Am J Phys Anthropol. 2007;132(4):605-621. doi: 10.1002/ajpa.20543

3. Andrews RM, Kubacka I, Chinnery PF, Lightowlers RN, Turnbull DM, Howell N. Reanalysis and revision of the Cambridge reference sequence for human mitochondrial DNA. Nat Genet. 1999;23(2):147. doi:10.1038/13779

4. Rohland N, Siedel H, Hofreiter M. A rapid column-based ancient DNA extraction method for increased sample throughput. Mol Ecol Resour. 2010;10(4):677-683. doi: 10.1111/j.1755-0998.2009.02824.x

5. Haak W, Forster P, Bramanti B, Matsumura S, Brandt G, Tanzer M, et al. Ancient DNA from the first European farmers in 7500-year-old Neolithic sites. Science. 2005;310(5750):1016-1018. doi: 10.1126/science.1118725

6. Handt O, Krings M, Ward RH, Pääbo S. The retrieval of ancient human DNA sequences. Am J Hum Genet. 1996;59(2):368-376.
